# Co-occurrence of anaemia and malaria among children under five in Uganda: A joint modeling analysis

**DOI:** 10.1371/journal.pgph.0007387

**Published:** 2026-10-01

**Authors:** Grace Kakaire, Robert Too, Gregory Kerich, Mathew Kosgei

**Affiliations:** 1 Department of Statistical Methods and Actuarial Sciences, School of Statistics and Planning, Makerere University, Kampala, Uganda; 2 Department of Mathematics, Physics and Computing, School of Science and Aerospace Studies, Moi University, Eldoret, Kenya; PLOS: Public Library of Science, UNITED STATES OF AMERICA

## Abstract

Childhood illnesses such as anaemia and malaria continue to pose significant health risks for children under five in sub-Saharan Africa. These conditions often co-occur, reflecting complex interdependencies driven by shared biological and environmental factors. Traditional analytical approaches typically assume independence between health outcomes, potentially overlooking meaningful associations. This study aims to identify the dependence structure among anaemia and malaria in children under five using a joint modeling approach. Data were drawn from the most recent Malaria Indicator Survey (MIS), comprising 5,755 children. A bivariate probit model was estimated using a Gaussian copula framework with probit margins for each outcome. This approach allowed for the simultaneous modeling of the marginal probability of each illness and the latent correlations among them. The model incorporated a range of child-, household-, and maternal-level covariates. Average Marginal Effects (AMEs) were calculated to facilitate interpretation. The copula correlation (ρ) between anaemia and malaria from the copula structure was estimated to quantify the dependence between the two illnesses. Descriptive analyses revealed substantial co-occurrence: 13.8% of children experienced both illnesses simultaneously. The joint copula model detected significant positive copula correlations (ρ ≈ 0.19) between anaemia and malaria. Several covariates displayed differential effects in the joint versus separate models. For instance, the association between maternal anaemia and child malaria risk reversed direction once interdependence was accounted for. Joint modeling yielded more stable and plausible estimates across outcomes. This study highlights the critical importance of accounting for outcome dependence in the analysis of child morbidity. The significant correlations among malaria and anaemia suggest that these illnesses are influenced by common or interacting risk factors. Joint modeling using a Gaussian copula framework enhances both the precision of marginal effect estimates and the understanding of comorbidity patterns. These findings support the design of integrated health interventions that address the interconnected nature of childhood illnesses in high-burden settings.

## Introduction

Childhood morbidity remains a major public health challenge in Uganda, where infectious diseases and nutritional deficits intersect in high-burden communities. National survey evidence shows that malaria infection remains common among under-five children; for example, analyses of the 2018–2019 Uganda Malaria Indicator Survey report an overall malaria positivity of ~21% among children under five (based on survey malaria testing) [1–4]. At the same time, childhood anaemia is highly prevalent: the 2016 Uganda Demographic and Health Survey reported that 53% of children aged 6–59 months had some degree of anaemia (24% mild, 27% moderate, 2% severe) [4]. Together, these figures underscore the continued population-level burden of malaria and anaemia and motivate analytical approaches that can study their co-occurrence and shared determinants [1,5]. Anaemia affects approximately half of children under five years of age in sub-Saharan Africa and remains a major contributor to childhood morbidity, impaired growth, and cognitive development. Its aetiology is multifactorial, reflecting the combined effects of malnutrition, infectious diseases (including malaria and other infections), and chronic inflammation, which frequently coexist in high-burden settings such as Uganda [4]. Malaria is a leading cause of child mortality in sub-Saharan Africa; according to the World Health Organization, children under five account for approximately 76% of malaria-related deaths in the region, underscoring its substantial contribution to under-five mortality [6]. These illnesses do not occur in isolation but often present simultaneously within the same child, resulting in what may be described as a syndemic pattern of disease—where multiple, interrelated conditions interact to amplify vulnerability and health risk [7].

Anaemia and malaria share multiple biological, environmental, and socioeconomic risk factors. Malaria infection contributes directly to anaemia through hemolysis and bone marrow suppression, while poor nutritional status and poverty increase susceptibility to both conditions [8]. Numerous studies have documented the high prevalence of each disease individually and have identified key determinants such as household wealth, maternal education, sanitation, and access to preventive and curative health services [2,3,9–11]. These studies have been instrumental in shaping disease-specific interventions [12] and policies aimed at reducing childhood morbidity [13].

However, what remains insufficiently understood is the dependence structure between anaemia and malaria at the individual level. Most epidemiological studies continue to analyze these conditions using separate regression models, implicitly assuming that the two outcomes are conditionally independent once observed covariates are controlled for [14]. This assumption is problematic in settings where diseases are biologically interconnected and influenced by shared unobserved factors, such as immunological vulnerability or environmental exposure. As a result, traditional univariate approaches may underestimate the true burden of comorbidity, obscure the extent of disease clustering, and limit the ability to design integrated health interventions [15–17]. Empirical evidence quantifying the latent correlation between anaemia and malaria—beyond shared observed risk factors—remains sparse, particularly among children under five in high-burden contexts [1].

Recent advances in global health research have emphasized the importance of addressing childhood multimorbidity using analytical frameworks that model multiple correlated outcomes simultaneously [11,12]. Multivariate statistical methods offer the ability to estimate marginal effects of covariates on each illness while also capturing residual dependence arising from shared or unmeasured determinants [2,13–15]. Despite their relevance, these methods remain underutilized in the study of childhood anaemia and malaria, leaving a critical gap in understanding how these illnesses cluster within individuals and communities [18,19].

The expectation and contribution of the current study is to address this gap by applying a joint modeling framework that explicitly captures the residual dependence between anaemia and malaria among children under five. Rather than examining each condition in isolation, we estimate the latent association between the two outcomes while adjusting for shared risk factors [20–23]. This study therefore aims to jointly model anaemia and malaria among children under five in Uganda, with the objective of estimating the extent of their dependence while accounting for shared risk factors.

By quantifying the extent of disease interdependence, the study contributes new insights into the clustering of childhood illnesses and supports the growing call for integrated approaches to child health [19]. The findings are expected to inform epidemiological research and policy by highlighting the need for health system responses that address comorbidities rather than isolated diseases. In high-burden, resource-limited settings, recognizing and modeling these dependencies is essential for designing effective, equitable, and child-centered interventions [24].

## Materials and methods

### Ethical statement

The study obtained approval from MEASURE DHS to use their data, but consent to participate was not necessary as this was a secondary analysis of non-identiﬁable, publicly available data. The researchers treated the DHS data conﬁdentially, and no attempt was made to identify individual women interviewed in the survey. All procedures were conducted in compliance with applicable guidelines and regulations

### Study area

This study is based on nationally representative data from Uganda, a high malaria transmission setting in sub-Saharan Africa characterized by substantial regional variation in disease burden, socioeconomic conditions, and access to healthcare services.

### Data source and sample design

Data used in this study were obtained from the 2018–19 Uganda Malaria Indicator Survey (MIS), which was conducted between December 2018 and January 2019 to align with peak malaria transmission. The UMIS collected information on a nationally representative sample of women of childbearing age (15–49 years)**.** The sample was stratified into fifteen different survey regions of the country [4]. A stratified two-stage sample design was used to select the sample during the survey. In the first sampling stage, a total of 320 clusters were chosen based on probability proportional to size from the Enumeration areas (EAs) covered in the 2014 National Population and Housing Census (NPHC). From these clusters, 236 were in rural areas and 84 in urban areas while 22 clusters were selected proportionate to size from the EAs covered in the refugee frame. The second sampling stage involved selecting 28 households from each cluster by equal probability systematic sampling. UMIS household and individual response rates are documented in the final UMIS report [20]; our analyses rely on the DHS/UMIS sampling weights which incorporate adjustments for non-response and unequal selection probabilities. We analysed children listed as de facto residents (0–59 months) with biomarker testing eligibility. Household clustering and stratification were retained in all design-based estimates. Missing outcome data were rare for malaria microscopy/RDT and haemoglobin, but any child with missing outcome(s) was excluded from analysis of that specific outcome.

All women of age 15–49 years in the sampled households, who were either permanent residents or visitors in the household on the night preceding the survey, were eligible for interview. Similarly, all children of age less than 5 years were eligible for anaemia and malaria testing. The analytic dataset was derived from two DHS/MIS standard files: The Household Members Recode (PR) file, which contains malaria rapid diagnostic test (RDT) and anaemia biomarker results, and the Children’s Recode (KR) file, which contains demographic and maternal/child background information. The unit of analysis was children under 5 years of age who were tested for malaria. Women 15–49 years were part of the MIS sampling frame but were not analyzed in this study. Descriptive statistics accounted for the complex survey design using DHS sampling weights, clustering, and stratification. Joint Gaussian copula regression models were estimated using the **GJRM** package in R. At present, this framework does not natively support survey-weighted likelihood estimation or full incorporation of complex survey design features. Consequently, the joint models were estimated without sampling weights, while design-based methods were retained for descriptive statistics and marginal benchmark models. To evaluate the robustness of inferences to this limitation, we conducted sensitivity analyses using survey-weighted marginal probit regressions and multilevel probit models with PSU-level random effects. These analyses yielded substantively similar results, supporting the stability of the main findings.

All statistical analyses were performed using R statistical software (version 4.4.2).

### Data linkage

The KR and PR files were merged using the household identifier (hhid), constructed from the KR file’s case id, to match the household IDs in the PR file. Mismatches, duplicates, and inconsistencies were checked for, during the merging process. Records with conflicting or missing household identifiers were carefully reviewed and excluded if linkage could not be confidently established. The final merged dataset was assessed for representativeness by comparing key demographic variables before and after merging to ensure no systematic bias was introduced due to linkage errors. Overall, 2.3% of records were excluded due to unresolved linkage inconsistencies. To assess whether linkage exclusions introduced systematic bias, key demographic characteristics—including child age group, sex, region, and place of residence—were compared before and after merging using χ² tests for categorical variables. No statistically significant differences were observed across these variables (all p > 0.05), indicating that the final merged dataset remained representative of the original survey population. A before-and-after linkage comparison table is provided in the Supplementary Materials (S1 Table)

### Data collection and measurements

Children under five were eligible for malaria testing, while anaemia testing was conducted among children aged 6–59 months, with parental or guardian consent. The joint model was fitted on children with non-missing malaria and anaemia measurements; because haemoglobin was measured among children aged 6–59 months, the joint-analysis sample is restricted to 6–59 months. Trained nurses were responsible for the testing and the children who tested positive were given medication on the spot, according to the national rules.

### Anaemia testing

Blood samples were collected from each child aged 6–59 months using a spring-loaded, sterile lancet to make a finger- or heel-prick. The drop of blood was collected in a microcuvette, and the Haemoglobin level analysed using a portable HemoCue analyzer. The results were available within 10 min and were given to the child’s parent or guardian verbally and in writing [20]. Parents were encouraged to take the children with haemoglobin level less than 11 g/dl to the nearest health care facility for the follow-up. Haemoglobin values used in this analysis were altitude-adjusted values provided in the MIS dataset, which account for geographic variation in elevation. The parents were given a referral letter with the haemoglobin examination to show the staff at the health care office. The results from the anaemia test were captured on the Biomarker Questionnaire and on the handout left in the household that contained information on the causes and prevention of anaemia [20].

Haemoglobin testing was not conducted among infants aged 0–5 months; therefore, estimates of anaemia prevalence and co-occurrence pertain only to children aged 6–59 months.

### Malaria testing

Malaria testing by microscopy involved preparing both the thick and thin blood smears using 2% Giemsa stain. As a quality control measure, each smear was read by two independent expert microscopists who were not involved with the participant respondent interview. A third reader was only involved to settle parasite presence discrepancy between the two readers. A positive smear parasite density (parasites/µl) was estimated by counting the number of asexual parasites per 200 white blood cells, assuming a white blood cell count of 8000 cells/µl. A smear was only considered negative if no parasites were seen after viewing 100 high-powered fields. Thin smears of positive thick smears were read to determine the species of Plasmodium parasite. Additionally, a malaria rapid diagnostic test was performed in order to offer prompt treatment to participants that were positive for malaria.

For this study, Malaria infection was defined as microscopy-confirmed parasitaemia (1 = positive, 0 = negative) because species-specific infections were relatively infrequent and because *P. falciparum* accounts for most malaria-related morbidity and anaemia in this setting. This binary specification is consistent with prior MIS-based studies and supports stable estimation within the joint modeling framework. As microscopy captures point-prevalence infection at the time of the survey, malaria status may not fully reflect recent or resolved infections.

### Sample selection and preparation

The analysis was restricted to de facto children under 5 years of age (0–59 months), i.e., those who were present in the household the night before the survey. Children without valid anaemia and/or malaria RDT or microscopy results were excluded. Specifically, of **7,124** eligible children, **4.03%** were excluded for missing outcomes and **15.2%** for missing covariates; the final analytic sample was **5,755** (**80.8%**) children, as a final analytical sample in order to ascertain a national-level representation [20]

### Defining variables and measurements

The study has two outcome variables of interest, namely

(i) having had malaria parasites by microscopy
(ii) child’s anaemia status [25]..

### Data structure

Let $Y_{ij}$ be the binary response for illness j∈{1,2} for individual i∈{1,...,n}, where:

- $Y_{i1}$ represents the presence of Anaemia
- $Y_{i1}$$Y_{i2}$ represents the presence of Malaria

Thus, for any child i, the response vector is ${𝐘}_{i}={\left(Y_{i1},Y_{i2}\right)}^{⊤}$.

Covariate vector ${𝐗}_{ij}$ includes the following variables for each outcome j (Table 1), organized in appropriate domains (Child-level, Maternal, Household-level):

**Table 1:** Summary of covariates and their categories.

Domain	Variable	Categories
Child-level	Child’s age	<1, 1 - < 2, 2 - < 5
Child-level	Child’s sex	Female, Male
Maternal	Mother’s Wealth status	Poor, Medium, Rich
Maternal	Mother’s education level	Tertiary, Secondary, Primary, No education
Maternal	Number of antenatal visits	>= 4, < 4
Maternal	Mother’s anaemia status	Anemic, Not anemic
Maternal	Mother’s age	<20, 20-39, 40+
Household-level	Residence status	Rural, Urban
Household-level	Type of toilet facility	Improved, Not Improved
Household-level	Type of water facility	Improved, Not Improved
Household-level	Region	Central, Others
Household-level	Number of children ever born	<= 2,> 2

These covariates help explain the likelihood of an individual experiencing one or more of these childhood illnesses.

### Conceptual diagram of the copula framework

The conceptual framework presented in Fig 1 illustrates the joint modeling approach used to analyze the co-occurrence of childhood illnesses—anaemia and malaria—based on the dataset. The framework decomposes the modeling process into marginal models for each binary outcome using probit regression, and then binds these via a Gaussian copula function to account for interdependence among the outcomes. Estimation is carried out using Maximum Simulated Likelihood (MSL), providing a robust strategy to jointly model correlated binary health outcomes while incorporating relevant covariates.

**Fig 1:**
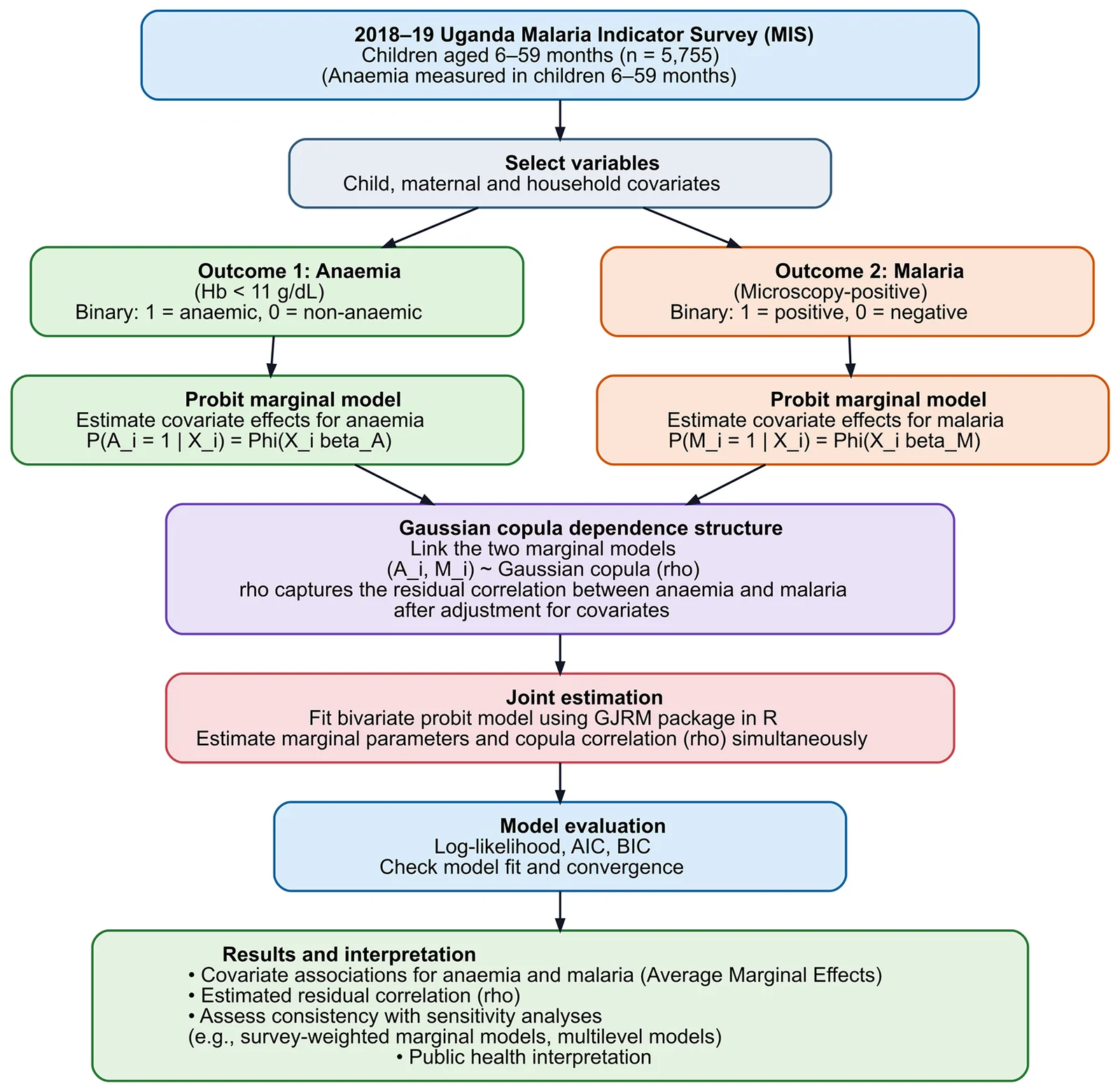
Flowchart summarizing the Gaussian copula joint modelling approach.

### Methodological flowchart

Fig 2 summarizes the methodological workflow, from data preparation and outcome definition to specification and estimation of the joint Gaussian copula model.

**Fig 2:**
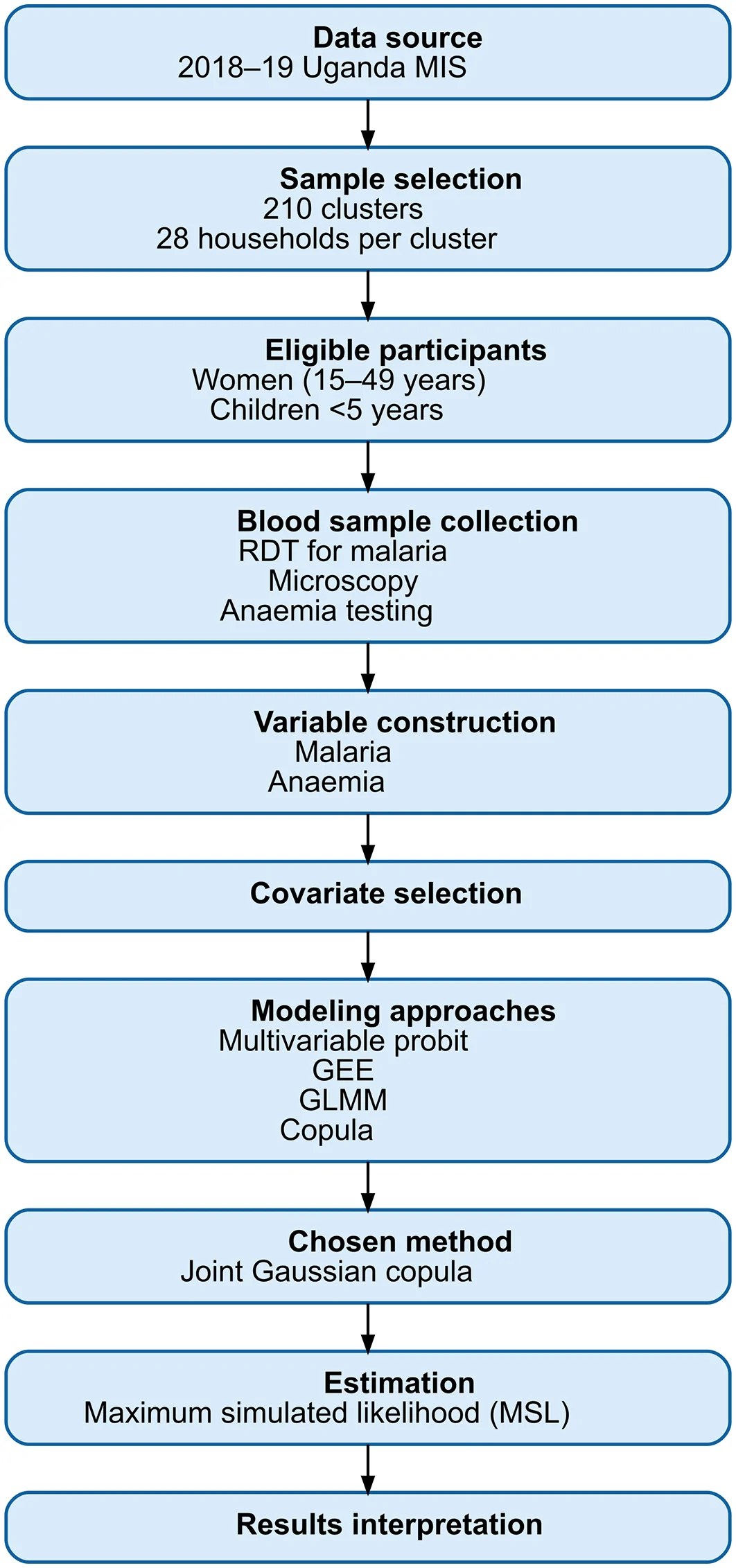
Flowchart summarizing the methodological approach.

### Statistical model and analysis

#### Joint models for correlated binary outcomes.

We jointly analyzed the occurrence of anaemia and malaria among children using a multivariate modeling framework designed for correlated binary outcomes. Because these conditions may co-occur within the same child due to shared biological, environmental, or socioeconomic factors, separate univariate models may yield inefficient or biased inference. We therefore modeled child health status as a bivariate binary outcome vector [26]


$$\begin{array}{c} Y_{i}=(Y_{i\text{1}},Y_{i\text{2}}{)}^{⊤}, \end{array}$$

where $Y_{i\text{1}}$ and $Y_{i\text{2}}$ indicate the presence of anaemia and malaria, respectively.

Each outcome was specified using a probit regression model. For outcome the marginal model was defined as

where $X_{ij}$ is the vector of covariates for child i and outcome j, $\beta_{j}$ is the corresponding vector of regression coefficients, and $\Phi^{-\text{1}}(\cdot )$ denotes the inverse standard normal cumulative distribution function [27].

#### Copula specification.

To capture dependence between anaemia and malaria beyond that explained by observed covariates, we employed a Gaussian copula to jointly model the two probit margins. The Gaussian copula allows the marginal distributions to be modeled separately while introducing a correlation structure that reflects unobserved factors influencing both outcomes. This approach is well suited to comorbidity settings where dependence is expected to be symmetric and not confined to extreme outcome probabilities.

The Gaussian copula is defined as [28]


$$\begin{array}{c} C(u_{\text{1}},u_{\text{2}};\Sigma )=\Phi_{\text{2}}(\Phi^{-\text{1}}(u_{\text{1}}),\Phi^{-\text{1}}(u_{\text{2}});\Sigma ), \end{array}$$

where $u_{j}=\text{Pr}(Y_{j}\leq y_{j})$ are the marginal cumulative distribution functions, $\Phi_{\text{2}}(\cdot ;\Sigma )$ is the bivariate normal cumulative distribution function with zero means and correlation matrix Σ, and the off-diagonal element of Σ represents the residual association between anaemia and malaria [5].

#### Estimation.

Model parameters were estimated using the gjrm() function from the GJRM package in R, specifying probit links for both marginal models and a Gaussian copula to characterize dependence. Because the outcomes are binary, estimation was conducted via maximum simulated likelihood [29]. Detailed derivations of the likelihood function and simulation procedure are provided in the Supplementary Methods (S1 File).

#### Interpretation: Average marginal effects.

To facilitate interpretation, we report Average Marginal Effects (AMEs) rather than raw probit coefficients. AMEs quantify the average change in the predicted probability of anaemia or malaria associated with a one-unit change in a covariate, averaging over the empirical distribution of all other covariates [30]. Computation of AMEs followed standard post-estimation simulation procedures; full details are provided in the Supplementary Methods (S2 File).

#### Model evaluation and robustness checks.

Model fit and predictive performance were assessed using the maximized log-likelihood, Akaike Information Criterion (AIC), and Bayesian Information Criterion (BIC). Residual dependence between anaemia and malaria was evaluated by testing the null hypothesis of zero copula correlation using a Wald test. For each marginal outcome, we computed Tjur’s $R^{\text{2}}$ and the area under the receiver operating characteristic curve (AUC) using predicted probabilities from the fitted joint model. For comparison, McFadden’s pseudo-$R^{\text{2}}$ values were obtained from separate survey-weighted probit regressions [30].

Robustness of the joint copula framework was examined using alternative modeling strategies, including generalized linear mixed models and survey-weighted marginal models. Results were consistent in magnitude and direction with those from the joint copula model. Full specifications, bootstrap procedures, and diagnostic checks are reported in the Supplementary Methods (S3 File).

### Ethical considerations

The study obtained approval from MEASURE DHS to use their data, but consent to participate was not necessary as this was a secondary analysis of non-identifiable, publicly available data. The researchers treated the DHS data confidentially, and no attempt was made to identify individual women interviewed in the survey. All procedures were conducted in compliance with applicable guidelines and regulations.

## Results

### Prevalence of malaria and anaemia

Among children with complete biomarker data (N = 5,755), 2,849 (49.5%; 95% CI: 48.3–50.7) had neither condition, 1,249 (21.7%; 95% CI: 20.7–22.7) had anaemia only, 863 (15.0%; 95% CI: 14.1–15.8) had malaria only, and 794 (13.8%; 95% CI: 13.0–14.7) had both malaria and anaemia (Table 2). These conditional prevalences reflect point-prevalence measurements of malaria and the multifactorial aetiology of childhood anaemia and are consistent with prior MIS-based studies in malaria-endemic settings.

**Table 2:** Prevalence of malaria and anaemia combinations.

Combination	n	Percentage	Prevalence CI
Neither anaemia nor malaria	2,849	49.5	48.289-50.673
Anaemia only	1,249	21.7	20.748-22.717
Malaria only	863	15.0	14.139-15.815
Malaria and anaemia	794	13.8	13.012-14.663
Total	5,755	100	

### Capturing residual dependence: The rationale behind joint Gaussian copula models

The results in Table 3 reveal that, the Gaussian copula model estimated a residual correlation of ρ = 0.191 (95% CI: 0.124–0.244) between anaemia and malaria, indicating a small positive latent association after adjustment for the observed covariates included in the two marginal models. This finding suggests that the two outcomes retain some dependence beyond that explained by the measured child-, maternal-, and household-level characteristics. Such residual dependence may reflect unmeasured biological, nutritional, environmental, or socioeconomic factors; however, the cross-sectional design does not permit identification of the specific mechanisms responsible for this association.

**Table 3:** Estimated correlation between anaemia and malaria from the Copula model.

Correlation	Estimate	CI
Anaemia-Malaria (θ_12_)	0.191	0.124 – 0.244

### Comparison of separate and joint marginal models for malaria and Anaemia

This section presents an interpretation of the results from both separate and joint marginal modeling approaches and highlights key differences in inference and implications.

#### Determinants of malaria: Separate and joint model comparison (Table 4).

The probability of malaria increases with the child’s age in both models, particularly for those aged one to under two years and two to under five years. This effect is more pronounced in the joint model, suggesting that controlling for illness interdependence sharpens the age-related association. Wealthier households consistently show a higher risk of malaria in both models, although the magnitude of this effect is slightly reduced in the joint model. A notable discrepancy is observed in the effect of maternal education. While the separate model associates secondary education with an increased risk of malaria, the joint model reveals a negative association, indicating that the effect observed in the separate model may be confounded by its association with other illnesses.

**Table 4:** Separate and marginal model for malaria.

Mother/Child Characteristic	Separate model		Joint model	
	Marginal Effect	P-value	Marginal Effect	P-value
(Intercept)	0.370	< 0.001	-0.125	< 0.001
Child’s age				
<1	Ref		Ref	
1 - < 2	0.056	0.019	0.451	0.022
2 - < 5	0.126	< 0.001	0.232	< 0.001
Child’s sex				
Female	Ref		Ref	
Male	0.003	0.828	-0.344	0.822
Mother’s Wealth status				
Poor	Ref		Ref	
Medium	0.068	0.006	0.366	0.006
Rich	0.178	< 0.001	0.208	< 0.001
Mother’s education level				
Tertiary	Ref		Ref	
secondary	0.145	0.022	-0.479	0.025
primary	0.068	0.267	0.470	0.297
no education	0.002	0.978	0.332	0.962
Number of antenatal visits				
>= 4	Ref		Ref	
< 4	0.008	0.664	-0.288	0.636
Mother’s anaemia status				
Anaemic	Ref		Ref	
Not anaemic	0.223	< 0.001	-0.318	< 0.001
Mother’s age				
<20	Ref		Ref	
20-39	0.023	0.524	-0.317	0.496
40+	0.030	0.449	-0.196	0.422
Residence status				
Rural	Ref		Ref	
Urban	0.128	< 0.001	0.025	< 0.001
Type of toilet facility				
Improved	Ref		Ref	
Not Improved	0.039	0.272	-0.068	0.305
Type of water facility				
Improved	Ref		Ref	
Not Improved	0.063	0.001	-0.209	0.001
Region				
Central	Ref		Ref	
others	0.110	< 0.001	0.112	< 0.001
Number of children ever born				
<= 2	Ref		Ref	
> 2	0.348	< 0.001	-0.134	< 0.001

Maternal anaemia status also shows divergent effects. In the separate model, children of non-anemic mothers appear more likely to have malaria, whereas the joint model reverses this relationship, indicating a protective effect of maternal non-anaemia when illness co-occurrence is considered. Regional, environmental, and household demographic factors remain significant across both models, although their effect sizes tend to be weaker or shift in direction under the joint modeling framework. Overall, the joint model indicates that the separate model may yield different estimates when outcome dependence is not accounted for.

#### Determinants of anaemia: Separate and joint model comparison (Table 5).

Children aged two to under five years are at significantly greater risk of anaemia in both models, and this association is stronger in the joint model. Secondary maternal education is associated with increased anaemia risk in both specifications, and the estimates are consistent across models. The number of children ever born in the household is also positively associated with childhood anaemia in both models.

**Table 5:** Separate and marginal model for anaemia.

Mother/Child Characteristic	Separate Model		Joint Model	
	Marginal Effect	P-value	Marginal Effect	P-value
(Intercept)	-0.246	< 0.001	-0.393	< 0.001
Child’s age				
<1	Ref		Ref	
1 - < 2	-0.005	0.783	-0.008	0.783
2 - < 5	0.070	0.003	0.112	< 0.001
Child’s sex				
Female	Ref		Ref	
Male	-0.013	0.327	-0.021	0.325
Mother’s Wealth status				
Poor	Ref		Ref	
Medium	-0.019	0.401	-0.031	0.401
Rich	0.014	0.692	0.023	0.693
Mother’s education level				
Tertiary	Ref		Ref	
secondary	0.126	0.008	0.117	0.008
primary	0.074	0.105	0.055	0.150
no education	0.034	0.446	0.055	0.142
Number of antenatal visits				
>= 4	Ref		Ref	
< 4	-0.013	0.407	-0.024	0.407
Mother’s anaemia status				
Anemic	Ref		Ref	
Not anemic	-2.102	0.890	-0.338	0.905
Mother’s age				
<20	Ref		Ref	
20-39	0.019	0.531	0.031	0.337
40+	0.011	0.740	-0.018	0.740
Residence status				
Rural	Ref		Ref	
Urban	-0.045	0.049	0.005	0.855
Type of toilet facility				
Improved	Ref		Ref	
Not Improved	-0.048	0.085	-0.077	0.088
Type of water facility				
Improved	Ref		Ref	
Not Improved	-0.031	0.057	-0.049	0.574
Region				
Central	Ref		Ref	
others	0.082	0.001	0.119	0.003
Number of children ever born				
<= 2	Ref		Ref	
> 2	0.190	< 0.001	0.140	< 0.001

Regional effects are consistently significant, with children outside the central region showing higher anaemia probabilities. A striking difference arises in the estimated effect of maternal anaemia.

One of the more interesting differences between the separate and joint models involved maternal anaemia status. The estimated association changed considerably once the joint model accounted for the residual dependence between childhood malaria and anaemia. This shift does not necessarily mean that the joint model “corrected” the estimate from the separate model. Rather, it shows that the association with maternal anaemia is sensitive to how the relationship between the two childhood outcomes is modeled. Here’s the thing: when outcomes are correlated, analyzing them separately may miss information contained in their dependence. Joint modeling provides a useful way to account for this relationship while estimating their associations with relevant risk factors. Still, because the data are cross-sectional, these results should not be interpreted as causal, nor can they explain why the magnitude or direction of the maternal anaemia association changed between the two models.

Overall, the comparison shows that some associations depend on whether the model accounts for the residual relationship between malaria and anaemia. Estimates for factors such as age and region remained fairly consistent across the separate and joint models, while others changed in magnitude or even direction. These differences do not necessarily mean that the joint estimates are more accurate. Instead, the joint approach offers something the separate models cannot: it estimates the remaining dependence between malaria and anaemia while also examining how measured risk factors relate to each outcome. This can be particularly useful when studying childhood conditions that often occur together. It doesn’t replace separate outcome-specific analyses, but it can reveal patterns of dependence that those analyses may overlook.

The joint model demonstrates that ignoring the correlations among childhood illnesses can lead to misleading conclusions. In contrast, multivariate modeling corrects for shared risk factors and provides a more comprehensive understanding of child health dynamics. As such, joint modeling is strongly recommended in settings where multiple comorbid conditions co-occur, especially in resource-limited environments where understanding interlinked health challenges is critical for effective intervention planning.

### Model validation

The joint bivariate probit (Gaussian copula) achieved a log-likelihood of −0.0043, with AIC = 118.01 and BIC = 510.82. The copula correlation was \u03c1̂ = 0.191; a PSU‑level, strata‑preserving bootstrap yielded a 95% CI [0.124, 0.244], indicating a positive residual dependence between anaemia and malaria. Outcome‑level performance was strong for anaemia and acceptable for malaria: Tjur’s R² = 0.606 (anaemia) and 0.232 (malaria); AUC = 0.943 and 0.798, respectively; Brier scores = 0.090 and 0.159 (lower is better). For comparison, separate (weighted) probit models gave McFadden’s pseudo‑R² (deviance‑based) of 0.599 for anaemia and 0.206 for malaria, indicating substantially stronger explanatory power for anaemia than malaria under independent modeling assumptions.

## Discussion

This study examined the co-occurrence and shared risk factors of anaemia and malaria among children under five using nationally representative data [5,31]. A Gaussian copula regression model with probit margins was utilized to simultaneously estimate the marginal effects of the crucial covariates on the individual diseases, along with the latent relationship between the outcomes simultaneously [32,33].

The descriptive findings indicated substantial overlap between the illnesses, with 14% of the children experiencing both illnesses simultaneously. These findings emphasize the syndemic nature of illnesses among the study population to support the use of analytical approaches that extend beyond single-outcome models [14,34]. The conditional prevalences were P(anaemia | malaria) = 47.994% and P(malaria | anaemia) = 38.889%. These overlaps are lower than some earlier Uganda and high-burden benchmarks that used different surveys and diagnostic modalities. For example, using Uganda MIS 2009 data, several studies reported substantially higher overlap, with 77.0% anaemia among malaria-positive children and 64.9% malaria positivity among anaemic children [7,32]. Similar patterns of stronger malaria–anaemia overlap have been observed in other high-transmission settings and time periods, where malaria contributes substantially to anaemia risk [8].

Several factors may explain the lower co-occurrence in our study. First, malaria and anaemia reflect different biological time windows: malaria positivity is a point-prevalence measure at the survey visit, whereas anaemia may persist for weeks to months after infection or arise from non-malarial causes (e.g., iron deficiency, helminths, inflammation), weakening contemporaneous overlap [31]. Second, case definitions and diagnostic modality can influence overlap estimates; DHS/MIS malaria measurement may be based on microscopy and/or RDT, and misclassification (including false-positive RDTs documented for the 2018–19 Uganda MIS) could shift estimated conditional prevalences [35]. Third, geographic heterogeneity and improvements in malaria control can reduce malaria-attributable anaemia while leaving substantial anaemia from competing causes, producing lower overlap even in endemic settings [7,34]. These considerations support interpreting co-occurrence in light of measurement choices and the multifactorial aetiology of childhood anaemia, and they further motivate our joint modeling framework to quantify residual dependence beyond observed covariates.

Unlike previous studies that model anaemia and malaria independently, this study provides empirical evidence of residual dependence between the two conditions after adjusting for shared covariates, using a copula-based joint modeling framework applied to nationally representative data [6].

The analysis showed that anaemia and malaria tend to occur together within the same child beyond shared observable risk factors [5]. A significant positive association between anaemia and malaria indicates that children affected by one condition are more likely to experience the other, even after accounting for known risk factors. This residual dependence is consistent with shared vulnerability due to unmeasured factors (e.g., nutrition, exposure, access to care) that may influence both outcomes, such as nutritional deficiencies, environmental exposures, or access to healthcare, which could affect multiple outcomes simultaneously [5,36]. The modest magnitude of the estimated residual correlation is therefore expected and should be interpreted in light of several biological and epidemiological considerations. Childhood anaemia is etiologically heterogeneous: although malaria contributes through hemolysis, inflammation, and bone-marrow suppression, a substantial share of anaemia in Ugandan children arises from non-malarial causes such as nutritional deficiencies, helminth infections, and chronic inflammation. These competing pathways attenuate the residual association between malaria and anaemia after covariate adjustment. In addition, the two conditions exhibit different age-specific and temporal patterns—malaria risk increases with cumulative exposure during early childhood, whereas anaemia is most prevalent in infancy and early toddlerhood and may persist after malaria clearance—leading to temporal mismatch in cross-sectional data and further weakening contemporaneous correlation despite an underlying biological link.

Comparing the joint model to the separate marginal regressions, the following salient findings resulted. A different and reasonable estimate of the effects, to begin with, resulted from the joint model compared to the separate model [9]. To illustrate, maternal anaemia was linked to lower risk of malaria under the joint model, while the separate model generated an intuitive opposite association. As well, the impact of maternal education on the risk of malaria flipped sign under the introduction of between-illness correlation into the separate model. These findings indicate that separate models could be subject to uncontrolled correlation between outcomes, producing misleading policy insights [37]. The reversal of the maternal anaemia–child malaria association under joint modeling likely yields attenuated estimates from unmeasured shared risk factors [38]. Maternal non-anaemia may proxy better nutrition, healthcare access, and preventive behaviors that jointly reduce malaria risk, while separate models conflate these pathways. By accounting for latent dependence, the joint model yields estimates that are more biologically and socially plausible [18,32].

With anaemia, the implausible large negative marginal effect of maternal non-anaemia that was witnessed in the separate model was greatly attenuated and made to be no longer significant in the joint model, with this serving to demonstrate how the omission of outcome dependence distorts results [39].

Considering both conditions together provides a more realistic picture of child health by capturing how illnesses cluster within individuals [40]. It further permitted the estimation of latent correlation structures between the outcomes, revealing insightful information regarding the clustering of illnesses at the individual level. These findings highlight the importance of studying multiple childhood illnesses together rather than in isolation [41].

From the perspective of public health, the potential benefit of integrated treatments that tackle the concurrent burden of the illnesses simultaneously. Single-disease policies and programs can fail to take the opportunity to target the co-determinants or the co-occurrence of the illnesses, thus reducing the potential effectiveness of the interventions. Statistically significant associations between the illnesses indicate common structural or biological vulnerabilities to be targeted by multifaceted, child-centered healthcare approaches [34].

The association between malaria and anaemia is supported by well-established biological and epidemiological mechanisms. Malaria contributes directly to anaemia through the destruction of red blood cells (haemolysis), both from parasite invasion and immune-mediated clearance of uninfected erythrocytes. In addition, malaria infection suppresses bone marrow function and impairs erythropoiesis, reducing the body’s ability to replace lost red blood cells. Inflammatory responses triggered by infection further disrupt iron metabolism and haemoglobin synthesis [42–44].

However, childhood anaemia in high-burden settings such as Uganda is multifactorial. Nutritional deficiencies (particularly iron deficiency), helminth infections, and chronic inflammation independently contribute to reduced haemoglobin levels. These overlapping causes mean that anaemia may persist even in the absence of active malaria infection [45].

From an epidemiological perspective, malaria and anaemia also differ in their temporal dynamics. Malaria infection reflects point prevalence at the time of measurement, whereas anaemia develops and persists over a longer period. This temporal mismatch can reduce observed co-occurrence in cross-sectional data, even when a strong biological relationship exists [46,47]. Together, these mechanisms help explain both the observed overlap and the modest residual association between the two conditions.

Differences observed between separate and joint analyses suggest that considering co-occurring illnesses provides a more accurate understanding of child health risks [6]. When residual dependence exists, separate models may yield biased or unstable estimates, including changes in effect magnitude or direction once shared latent risk is accounted for [7].. By contrast, the joint copula approach estimates marginal effects and cross-equation dependence simultaneously, improving efficiency and interpretability [7]. This behavior is well documented in the multivariate probit and copula literature and supports our preference for a joint specification [10]. Our observed differences, attenuation and occasional sign changes (e.g., maternal education, maternal anaemia), are therefore consistent with theory when correlated outcomes are modeled jointly rather than separately.

The generalizability of these findings should be interpreted in light of Uganda’s marked geographic and seasonal heterogeneity in malaria transmission and anaemia burden. Malaria risk varies substantially across regions due to differences in transmission intensity, altitude, climate, and vector ecology, while anaemia prevalence reflects additional spatial variation in nutrition, helminth exposure, and socioeconomic conditions. The MIS was conducted during the peak transmission season, and malaria was measured as point prevalence; consequently, the observed co-occurrence and dependence may differ in lower-transmission seasons or in regions with distinct epidemiological profiles. Nevertheless, the nationally representative design supports broad inference at the population level, and the joint modeling framework remains applicable across regions and seasons, even though the magnitude of co-occurrence and residual dependence may vary contextually.

One issue to consider when interpreting the residual association between malaria and anaemia is that the two measurements capture different time periods [2,13–15]. Malaria microscopy detects parasitaemia present at the time of the survey, providing a snapshot of current infection. Haemoglobin concentration, by contrast, can reflect physiological changes that develop and persist over a longer period. A child affected by a recent malaria infection, for example, may still have reduced haemoglobin even after parasitaemia is no longer detectable. Conversely, a child with a current malaria infection may not yet have developed measurable anaemia. This mismatch in timing could weaken the association observed at a single survey visit. It’s therefore more appropriate to interpret the estimated residual correlation as the conditional association between malaria and anaemia at the time of measurement, rather than as a complete measure of their biological relationship over time.

This study has several limitations. First, anaemia was assessed only among children under five years, excluding younger infants. Also, malaria status was based on microscopy-confirmed parasitaemia, which reflects infection at a single point in time; anaemia may persist for weeks or months following malaria clearance, potentially attenuating contemporaneous overlap between the two conditions. As with most household-based surveys, children who were severely ill or hospitalized at the time of data collection may have been underrepresented, potentially resulting in conservative estimates of disease prevalence and co-occurrence.

Second, although the joint model captures residual correlation through latent dependence, some unobserved confounders—such as seasonal variability, nutritional status, or healthcare access—were not available in the dataset and may bias estimates.

Third, the assumption of symmetric dependence embedded in the Gaussian copula may not fully capture extreme patterns of co-infection or asymmetric disease dynamics, especially in subgroups such as rural or malnourished populations.

Fourth, Childhood anaemia has many causes, so the residual association observed in this study needs to be interpreted within that broader context. Malaria is only one contributor. Iron and other micronutrient deficiencies, helminth infections, inflammation, and other infectious conditions can also affect haemoglobin levels, yet the MIS did not directly measure many of these factors. This leaves some potentially important pathways unaccounted for in the current model.

Finally, while the model is statistically rigorous, it is computationally intensive and may not be easily implemented in low-resource settings without access to advanced statistical expertise or computing infrastructure.

To address the limitations identified and build on the study’s strengths, we recommend the following:

i Longitudinal Data Collection: Future studies should incorporate panel or cohort data to enable causal inference, establish temporal relationships, and examine the progression and persistence of co-infections over time.
ii Inclusion of Contextual Variables: Incorporating additional variables such as nutritional status, immunization history, healthcare access, and seasonal factors would provide a more comprehensive understanding of disease dynamics and help adjust for residual confounding.
iii Exploration of Alternative Copula Structures: While the Gaussian copula offers a symmetric dependence structure, future analyses should consider alternative copulas (e.g., Clayton, Gumbel) that can capture asymmetric tail dependencies and better reflect high-risk clustering in vulnerable subpopulations.
iv Validation with Objective Measures: Where feasible, repeated or longitudinal biomarker assessments and health facility-based diagnoses should be used to validate survey-reported outcomes and reduce measurement error.
v Model Simplification for Policy Use: To facilitate the use of joint modeling techniques in applied settings, simplified or modular versions of the model can be developed, along with user-friendly tools or dashboards for health system planners and program managers.
vi Future studies could build on this work by incorporating nutritional and inflammatory biomarkers, micronutrient indicators, and data on parasitic infections when these measures are available. Doing so could help separate malaria-related pathways from other causes of anaemia and give a clearer picture of how these conditions occur together in children. It’s an important direction for future research, particularly in settings where several nutritional and infectious risks overlap.
vii Capacity Building in Data Science and Analytics: Investments in local capacity for advanced statistical modeling will enhance the ability of national health institutions to routinely analyze and interpret complex data, thereby strengthening surveillance and intervention strategies.

### Methodological and statistical implications of our findings

The results of this research have significant statistical and methodological implications for modelling childhood comorbidities, especially for high-burden environments like Uganda. Classical models, under which individual illnesses are modeled separately, rely on the assumption of conditional independence between illnesses given observed covariates. Our results offer clear evidence that the assumption is not met for anaemia and malaria. The illnesses often co-occur and show substantial latent associations, as described by the joint Gaussian copula model.

Statistically, this illustrates how individual univariate probit or logistic models can generate inefficient or even biased marginal effects estimates if the outcomes are inherently correlated. In our analysis, for example, maternal anaemia and wealth index at the household level yielded opposite effects under individual and combined model specification, suggesting that not considering outcome interdependence can mislead substantive interpretation. In contrast, the joint modeling approach estimates simultaneously the marginal effects, correcting for the shared variation caused by unobserved factors operating on more than one outcome. The double estimation enhances statistical efficiency as well as the accuracy of inference.

Methodologically, the research showcases the benefits of using copula-based joint models for public health research. Copulas present a versatile methodology for separating marginal distributions from dependence structures, thus being ideal for multivariate binary data. The employed Gaussian copula is symmetrical and accommodates moderate to high associations between outcomes without assuming tail dependence, consistent with the pattern of comorbidity among children’s health. Additionally, applying simulated maximum likelihood for model estimation shows the potential for using computationally demanding approaches when applied to nationally representative surveys. The potential reaches beyond this single application. For situations involving many binary outcomes being aggregated — for example, household health surveys, demographic surveillance systems, or integrated disease surveillance systems — joint-modeling approaches may present a richer view of disease burden, association, and clustering. This is especially important for informing integrated interventions, maximizing resource allocation, and enhancing targeting of high-risk populations.

### Strengths of this study

This study provides a robust and nuanced analysis of co-occurring childhood illnesses in Uganda, leveraging nationally representative data from the Malaria Indicator Survey (MIS). The use of a joint modeling framework based on a Gaussian copula enables the simultaneous analysis of anaemia and malaria—two highly prevalent and interrelated conditions among children under five. Unlike traditional univariate approaches, this framework accounts for residual dependence between outcomes, yielding more reliable marginal effect estimates and allowing for more accurate insights into comorbidity patterns.

Furthermore, the inclusion of a wide range of covariates—spanning demographic, maternal, environmental, and household factors—enhances the explanatory power of the model. The use of Average Marginal Effects (AMEs) improves the interpretability of results, making them more relevant for public health practitioners and policymakers. The methodological rigor of the joint approach also strengthens the internal validity of the findings and sets a precedent for future analyses of multi-outcome health data in similar contexts.

Despite its contributions, the study has several limitations. First, the cross-sectional design of the MIS data restricts the ability to infer causality between covariates and health outcomes. The directionality of associations cannot be established, and reverse causation cannot be ruled out. Further, because this study is a secondary analysis of cross-sectional MIS data, several measurement constraints should be noted.

## Conclusion

In conclusion, this research illustrates the applicability of the joint Gaussian copula models to the analysis of comorbid childhood diseases. By capturing interdependencies while delivering interpretable marginal effects, the approach presents an efficient tool to comprehend, if not tackle, the multifaceted nature of health issues. Under the scenario where comorbidity is common, joint modeling needs to be implemented as the standard analytic practice to guide more integrated and efficient policy interventions.

## Supporting information

S1 Table Comparison of key characteristics before and after data linkage. Distribution of selected demographic characteristics before and after merging the KR and PR files, used to assess potential linkage-related bias.
(DOCX)

S2 Table Multicollinearity diagnostics (variance inflation factors). Variance inflation factors (VIFs) for all explanatory variables included in the joint Gaussian copula regression model, along with the mean VIF, used to assess multicollinearity.
(DOCX)

S1 File Maximum Simulated Likelihood estimation for the Gaussian copula model. Detailed derivation of the likelihood function, simulation algorithm, and numerical optimization procedures used to estimate the joint model.
(DOCX)

S2 File Computation of average marginal effects. Formal definition and computational details for estimating average marginal effects from the joint Gaussian copula model.
(DOCX)

S3 File Sensitivity analyses and robustness checks. Description of alternative model specifications, including survey-weighted marginal probit models, multilevel probit models with PSU-level random effects, and bootstrap procedures.
(DOCX)

S4 File Supplementary results: preliminary evidence of dependence between anaemia and malaria. Results from log-linear models assessing dependence between anaemia and malaria prior to joint regression modeling.
(DOCX)

S5 File Supplementary results: sensitivity analyses and model validation PSU-level random effects, and bootstrap procedures. (DOCX)

S6 File R code used for data management and statistical analysis. R scripts used for data cleaning, variable construction, estimation of separate and joint models, computation of average marginal effects, diagnostic checks, and simulation-based validation.
(R)
