# Supplementary material for: Co-occurrence of anaemia and malaria among children under five in Uganda: A joint modeling analysis
Source: PLOS Glob Public Health. 2026 Oct 1;6(10):e0007387. doi: 10.1371/journal.pgph.0007387 (PMC13630190; doi:10.1371/journal.pgph.0007387)
Supplement: Supplementary file 1 [file pgph.0007387.s010.pdf]

General Comments

Thank you for inviting me to review a manuscript entitled **"Co-occurrence of Anaemia and Malaria Among Children Under Five in Uganda: A Joint Modelling Analysis"**. This is an interesting study, and the authors have collected a good dataset using the critical advantage methodology. The paper is generally well written and structured. However, from my point of view, the manuscript has some shortcoming with respect to some dataset analyses and text, and I feel this good dataset has not been used to its full extent. Underneath, the reviewer has provided several remarks on the text, as it is often vague and long-winded. In several examples, I also recommended citing more relevant and recent literature. Moreover, the reviewer has made additional comments for more in-depth analyses of the dataset. I have assessed the manuscript, and I can hardly suggest it for publication for the following reasons:

Key critical points are:

a) Please check the grammar carefully in the overall manuscript; somewhere it's written in the past and somewhere in the present and some here in starting in the paragraph in small letter and also some here written capital letter in the middle. Check the spelling mistakes; there are major spelling errors in the overall document. If possible, please I suggest that this manuscript be reviewed by an English-language expert.

Specific Comments

1. The **Background section on the body of manuscript**: this section needs revision so that it will cover the nature of the core problem, the known aspect of problem (i.e., what is known about the core problem), the unknown aspect of the problem (What is unknown about the core problem), and the expectations from the current study (what and how the current study will add to the existing knowledge).
2. What type of statistical software do you use (i.e., R, STATA, SAS…) in your analysis and please upload as supplementary material the codes/commands of the statistical software?
3. In page 7 and line 130-131"Children aged 0–59 months were tested for both anaemia and malaria with the parents' or guardians' consent"? is Children aged 0–59 months??? Is it possible?
4. Please use the latest reference rather than old reference! Like reference 2, 3, 21 etc.…
5. Please include as a Table the shows the multicollinearity assumption with its mean of VIF test of the variables in supplementary materials?
6. Please approximate the results of tables of the analysis in to 3 digits of the result section? like 0.002, 1.12 etc.…
