# Supplementary material for: Co-occurrence of anaemia and malaria among children under five in Uganda: A joint modeling analysis
Source: PLOS Glob Public Health. 2026 Oct 1;6(10):e0007387. doi: 10.1371/journal.pgph.0007387 (PMC13630190; doi:10.1371/journal.pgph.0007387)
Supplement: Supplementary file 2 [file pgph.0007387.s012.pdf]

Note on the manuscript entitled (Co-occurrence of Anaemia and Malaria Among Children Under Five in Uganda: A Joint Modeling Analysis)

Abstract

- **Line 21:** Please Use "aims" in your sentence: "This study seeks to identify..." because it is more standardized in scientific research and is more appropriate in the epidemiological context.

Introduction

- **Lines 43-45:** The paragraph provides a description of the current situation in the research area, but it does not provide any statistical figures or research evidence to support this claim. It would be better to rephrase it and add figures that support this claim, whether from the World Health Organization, the Ugandan Ministry of Health, or even from any reliable and credible published research.
- **Lines 45-48:** The paragraph explains that anemia affects about half of children under five in the region, and the authors link this to malnutrition, chronic infections, and parasitic diseases. However, there are other causes such as bacterial and viral infections, so 'infectious diseases, including malaria' would have been better for greater comprehensiveness.
- **Lines 74-76:** This paragraph lacks a reference. Please add a reliable reference.
- **Lines 80-83:** The paragraph is more suited to the methodology section, so it should be moved there. It was also noted that the paragraphs at the end of the introduction lack references and are generally inconsistent. This should be reviewed and addressed as needed.
- The paragraphs from **line 77** to the end of the introduction aim to highlight the importance and purpose of the study. I think this is too much; these paragraphs need to be rewritten and shortened as much as possible because, in general, the introduction is long and.

- The introduction should be revised to incorporate information on malaria and anemia, their association, and the identification of risk factors in Uganda, as well as statistics from trustworthy sources such as the World Health Organization and the Ugandan Ministry of Health. The study's objectives and justifications should also be briefly stated.

Methodology

- The paragraphs are rich in information but lack a smooth flow from study design to statistical analysis.
-
- **It would be better to divide the section into clear headings:**
-
- Study area
-
- Data Source and Sample Design
-
- Sample Selection and Preparation
-
- Defining Variables and Measurements
-
- Statistical Model and Analysis
-
- Ethical Considerations
-
- The scientific approach is good, but it needs a greater focus on rationale rather than minor technical details.
- **Lines 92-93:** The UMIS 2018-2019 statistics are the most recent national survey data on malaria and anemia in Ugandan children. However, the fact that several years have elapsed since the survey may restrict the data' applicability to the present epidemiological situation, particularly in light of potential changes in control measures and disease burden.
- **Lines 92-119:** The description of the data source in **paragraphs 92–119** is overly detailed and lengthy, combining seven separate topics (data source,

sample design, weights, response rates, handling missing data, file merging, and unit of analysis) into a single, disorganized paragraph. This makes it difficult to read and violates the scientific brevity standards outlined in the Methodology section. It would have been better to summarize the survey description in 3–5 concise sentences, transferring technical details to subparagraphs or a table, to enhance clarity and focus on the study's relevant aspects.

- **Lines 121- 126:** The description of data linking in paragraphs 121–126 is technically clear but overly brief and lacks supporting quantitative evidence, thus diminishing its scientific credibility. While mentioning screening, exclusion, and representativeness verification, it fails to specify the percentage of records excluded, the statistical tests used to compare demographic variables (such as χ^2 or t-test), or the main variables compared, rendering the claim of "no bias" quantitatively unsupported. Adding a sentence containing figures (e.g., "2.3% of records were excluded, and no significant differences were found in age or region $p>0.05$") or a before/after linking comparison table would have been preferable to enhance transparency and methodological reliability.
- **Lines 28-131:** The ethical statement in paragraphs 128–131 is standard and entirely acceptable in terms of content and wording, covering the consent to use DHS public data, the absence of participant consent, confidentiality, and non-identification, while adhering to regulations. However, it lacks procedural precision due to the omission of the MEASURE DHS permit number, and the phrase 'women respondents' is inaccurate here, as the analysis focuses on children. It would have been better phrased as, 'The research has obtained MEASURE DHS (Permit No. XXX) approval to use public, non-identifiable data, while maintaining full confidentiality and complying with the Helsinki/DHS guidelines,' to enhance professional transparency.
- The section on **malaria testing** is very long and contains tedious details; it should be shortened to include only the important points.
- **Lines 161-162**: It is best to have complete consistency and coordination in the research, mentioning either 5 years of age or (0–59 months).
- **Lines 188–192:** The flowchart description in paragraphs 188–192 is redundant and unnecessary, repeating the same ideas three times without adding value,

thus weakening the impact and duplicating previous details. It would be better to shorten it to the sentence: “Figure 2 presents the flowchart’s systematic approach from data collection to Gaussian cupola model estimation, including model selection criteria,” while ensuring the figure is available.

- **Lines 192-362**: Excessively long and tedious: >150 lines with repetition of fundamental equations and concepts (Y, probit).
- **Unfocused:** A lengthy overview of GEE/GLMM without application or results.
- **Incomplete equations:** Cut off (9–10), without explanation of S in MSL.
- **Distracted checks**: VIFs/GLMM/bootstrap without preliminary results.
- **Solution:** Divide into 3 sections (justification, equations, evaluation) + appendices/comparison table.
- **In general, the methodology** is too long, boring the reader with repetition and unnecessary details. Excessive length, repetition, and detailed information discourage reading and bore the reader. It is recommended to be concise, mentioning only the essential points and those worthy of mention, integrating ideas, and refining the methodology.

Results

- **Lines 356-370** contain the same text as the table above, therefore the table is unnecessary as it is redundant and adds nothing new.
- **Lines 371-390:** The section is overly detailed and unfocused, repeating the log-linear equation twice (17, 18) with an unnecessary supplementary version (=7.845−1.568A−1.169M+0.971AM), and presenting a detailed table without full statistical explanation (such as AIC/BIC for comparison, or how to interpret OR=2.64 in the context of the study). The reference to [the relationship] is good, but it lacks mention of sample size or the fit test. It would have been better to summarize it in a short paragraph: "The homogeneous log-linear model showed the best fit (Table 3), with a significant A×M interaction (OR=2.64, p<0.001), confirming the dependency between anemia and malaria before the transition to the copula.
- **Lines 393-411:** Paragraphs 393–411 are repetitive and out of context, as they reiterate the “Why Coppola?” (conditional independence, residual correlation, margin comparison) previously mentioned in the methodology, while promising “results” (residual correlation, correlation value) that are not

presented here, resembling a “results introduction” rather than a justification. It would have been better to: **Transfer them to the methodology as a “preliminary reliability test prior to evaluation.**

- **Lines (419–455):** are overly detailed and lacks conciseness. It repeats the interpretation of variables across Tables (5, 6) without specific quantitative figures (such as AMEs or p-values), forcing the reader to refer back to the tables and weakening persuasiveness. Out of context: It reads more like a "discussion" than a "results," with explanatory phrases ("more realistic," "missing variable bias") added later. Unbalanced: It focuses on "differences" without providing the ρ correlation or its significance test, and ends abruptly. A better approach would have been: a concise comparison table of the main AMEs + a summary paragraph: "The combined model showed modifications in effects (e.g., maternal education, maternal anemia), underscoring the importance of dependency (Tables 5–6)," with the full explanation of the discussion being transferred.
- **Lines 470-514:** Inappropriate placement: The simulation is assigned to the "methodology" or appendices, not the main results; the final statistics (logL, AIC, ρ=0.191, AUC) are appropriate here but ultimately omitted. Lack of transparency: "Strong support" is claimed without evidence (bias, CI coverage), and comparisons are made with separate models without formal testing (Wald for ρ). A better approach would have been to move the simulation to the appendices and summarize the validation in a table: "The combined model succeeded (ρ=0.191, 95% CI[0.114, 0.263]; AUC=0.94 anemia, 0.80 malaria; Table X).
- The results are scattered and lengthy, needing to be organized. There is confusion between what belongs in the results section and what should be in the methodology or discussion section.

Discussion

- **Lines(520–527):** is a strong contribution, but it is vague and unsupported because it does not mention previous comparative references (“Unlike previous studies”), which weakens the claim and makes the reader search for them.

- **Lines 527-530:** Unsupported assumptions: “Common factors such as malnutrition…” are offered as an explanation without linking to the study results or additional evidence.
- The concluding paragraph **(543–549)** is a good summary of the benefits of the model, but it is general and not supported by specific evidence.
- **Lines 566- 591**: Excessive repetition: It repeats the previously mentioned "false conditional independence," "univariate bias," and "coupola benefits" dozens of times. Vague examples: "adverse effects of maternal anemia and wealth" without AME figures or significance tests for differences between models. Abrupt break: It ends with "accommodates moderate to high associations" without a conclusion.
- The “Strengths and Limitations” section (**593–636)** is excessively long and undefined (more than two pages), repeating previously known benefits of the model (“higher accuracy, AMEs”), with very general suggestions (longitudinal, alternative copulas) without direct link to the study results.

Dr. Zuhair Dardona
