# Supplementary material for: Co-occurrence of anaemia and malaria among children under five in Uganda: A joint modeling analysis
Source: PLOS Glob Public Health. 2026 Oct 1;6(10):e0007387. doi: 10.1371/journal.pgph.0007387 (PMC13630190; doi:10.1371/journal.pgph.0007387)
Supplement: Supplementary file 3 [file pgph.0007387.s016.pdf]

Note on the manuscript entitled (Co-occurrence of Anaemia and Malaria Among Children Under Five in Uganda: A Joint Modeling Analysis)

1. **Lines 52-57.** The paragraph requires clarification in several areas. First, the statement that malaria is one of the leading causes of child mortality should be supported with recent epidemiological data from reliable sources (e.g., WHO). Second, the distinction between infectious diseases and parasitic infestations is conceptually redundant, as parasitic infections are part of infectious diseases; the wording should be revised accordingly. Finally, the sentence structure describing the multifactorial aetiology of anaemia needs grammatical correction and clearer formulation.
2. **Lines 61-64.** The sentence "What is known from existing literature is that…" is stylistically weak and unnecessarily indirect for scientific writing. Academic writing typically favors more direct statements supported by citations.
3. **Lines 64-66**. The authors state that "Numerous studies have documented the high prevalence of each disease individually and have identified key determinants such as household wealth, maternal education, sanitation, and access to preventive and curative health services." However, no specific references are provided to support this statement.
4. **The introduction** should be revised to incorporate information on malaria and anemia, their association, and the identification of risk factors in Uganda. The study's objectives and justifications should also be briefly stated.

Methodology

The paragraphs are rich in information but lack a smooth flow from study design to statistical analysis.

-It would be better to divide the section into clear headings:

-Study area
-Data Source and Sample Design
-Sample Selection and Preparation
-Defining Variables and Measurements

-Statistical Model and Analysis

-Ethical Considerations

- The scientific approach is good, but it needs a greater focus on rationale rather than minor technical details**.**

In general, the methodology is too long, boring the reader with repetition and unnecessary details. Excessive length, repetition, and detailed information discourage reading and bore the reader. It is recommended to be concise, mentioning only the essential points and those worthy of mention, integrating ideas, and refining the methodology.

Results

1- **Line 268**. The manuscript reports that "21.720% had anaemia only", but the total number of participants corresponding to this percentage is not provided. In epidemiological reporting, percentages should always be accompanied by the absolute number of cases (n) and the total sample size (N) to allow readers to properly interpret the magnitude of the finding.

2- **Table 2 (Prevalence of Malaria and Anaemia Combinations)** appears to present information that has already been described in detail in the preceding text. This results in unnecessary redundancy and does not add additional analytical value. In scientific reporting, tables should provide new insights, clearer comparisons, or more detailed data that cannot be efficiently presented in the narrative text.

3- **Lines 287-282.** The paragraph beginning with "As discussed, we fitted two sets of models: separate marginal probit regressions and a joint Gaussian copula regression model" includes methodological explanations that are not appropriate for the Results section. The Results section should focus strictly on presenting the findings of the analysis, without reiterating methodological details or providing interpretation of the modeling strategy. Statements describing the modeling framework, assumptions (e.g., conditional independence), and the rationale for using a joint copula model belong in the Methods section, while interpretation of the findings should appear in the Discussion.

4- **Lines 283-287.** The paragraph beginning with "To assess whether a joint modeling approach was necessary…" contains methodological reasoning and justification for selecting the joint Gaussian copula regression model. Such

explanations are more appropriate for the Methods section, rather than the Results section.

5- **Lines 288-297**. The same above mentioned note.

6- **Lines 305-310.** The same above mentioned note.

7- The Results section currently shows substantial weaknesses in the clarity and scientific structure of result reporting. The presentation of findings is often mixed with methodological explanations, interpretations, and descriptive commentary, which makes the section difficult to follow and inconsistent with standard scientific reporting practices. In a well-structured manuscript, the Results section should present the empirical findings in a clear, concise, and neutral manner, without methodological repetition or interpretative statements. Furthermore,. In several instances, the tables simply repeat information already described in the text rather than providing additional insights or clearer organization of the data.

Discussion

1- Line 362. The phrase “In this paper, this study…” is redundant and stylistically weak. The use of both paper and study conveys the same meaning and results in unnecessary repetition.

2- Lines 368-377. The paragraph presents an extensive restatement of the numerical findings (e.g., percentages and conditional prevalences) that have already been reported in the Results section. In the Discussion section, it is generally unnecessary to repeat detailed statistics. The Discussion should briefly summarize the key findings and then focus on interpreting these findings, explaining possible mechanisms, and comparing them with previous studies. In its current form, the paragraph reads more like a continuation of the Results section rather than a critical interpretation of the findings.

In general, the most important observations on the discussion can be summarized as follows:

1- The discussion repeats too many results that were already presented in the Results section. The authors should summarize only the most important findings.
2- The section focuses too much on explaining the statistical model instead of discussing the public health meaning of the findings.
3- There is limited comparison with previous studies. The authors should clearly explain how their findings agree or differ from earlier research.
4- Some results are mentioned without providing clear explanations for why they occurred.
5- The discussion does not clearly explain the biological or epidemiological mechanisms linking malaria and anaemia.
6- The practical implications of the findings for public health policy or interventions are not sufficiently discussed.
7- The limitations of the study are not clearly presented in the discussion.
8- The discussion structure is not well organized and should follow a clearer flow (main findings → interpretation → comparison with literature → implications → limitations).
