# Supplementary material for: Co-occurrence of anaemia and malaria among children under five in Uganda: A joint modeling analysis
Source: PLOS Glob Public Health. 2026 Oct 1;6(10):e0007387. doi: 10.1371/journal.pgph.0007387 (PMC13630190; doi:10.1371/journal.pgph.0007387)
Supplement: Supplementary file 4 [file pgph.0007387.s001.docx]

| **S1 Table. Comparison of Key Characteristics Before and After Data Linkage** | | | | |
| --- | --- | --- | --- | --- |
| Characteristic (Children 6–59 months) | Pre-linkage Sample (N = 5,891) | Post-linkage Analytic Sample (N = 5,755) | Statistical Test | p-value |
| Age group distribution | Similar proportions | Similar proportions | χ² test | > 0.05 |
| Sex (male/female) | No meaningful difference | No meaningful difference | χ² test | > 0.05 |
| Region | Comparable across regions | Comparable across regions | χ² test | > 0.05 |
| Residence (urban/rural) | Comparable | Comparable | χ² test | > 0.05 |

**The pre-linkage sample includes all children with valid KR or PR records prior to file merging.*

**The post-linkage analytic sample excludes 2.3% of records due to unresolved household identifier mismatches or missing linkage information.*

**No statistically significant differences were observed between pre- and post-linkage samples across key demographic characteristics, indicating no evidence of linkage-induced selection bias.*
