# Supplementary material for: Co-occurrence of anaemia and malaria among children under five in Uganda: A joint modeling analysis
Source: PLOS Glob Public Health. 2026 Oct 1;6(10):e0007387. doi: 10.1371/journal.pgph.0007387 (PMC13630190; doi:10.1371/journal.pgph.0007387)
Supplement: Supplementary file 5 [file pgph.0007387.s002.docx]

**Supplementary Methods: Assessment of Multicollinearity**

Multicollinearity among the explanatory variables included in the joint model was assessed using Variance Inflation Factors (VIFs). VIFs were computed from an auxiliary linear regression model that included the full set of covariates used in the joint analysis. This approach is standard practice for evaluating linear dependence among predictors, even when the primary models of interest are non-linear (e.g., probit models).

Multicollinearity was evaluated using commonly accepted criteria, where VIF values below 5 indicate no serious multicollinearity.

**Supplementary Results: Multicollinearity Diagnostics**

The results of the multicollinearity assessment are presented in **Supplementary Table S2**. All adjusted VIF values were well below the conventional threshold of concern. The mean VIF across all covariates was 1.103, indicating no evidence of problematic multicollinearity among the explanatory variables included in the joint model.

| **S2 Table. Variance Inflation Factors (VIFs) for Covariates Included in the Joint Model** | |
| --- | --- |
| **Variable** | **VIF** |
| Child's age | 1.025 |
| Child's sex | 1.003 |
| Mother's Wealth status | 1.151 |
| Mother's education level | 1.091 |
| Number of Antenatal Visits | 1.04 |
| Mother's Anemia Status | 1.063 |
| mother's Age | 1.097 |
| Residence status | 1.252 |
| Type of Toilet Facility | 1.19 |
| Type of Water Facility | 1.071 |
| Region | 1.129 |
| Number of children ever born | 1.205 |
| Religion | 1.026 |
| Mean VIF | 1.103 |
