# Supplementary material for: Co-occurrence of anaemia and malaria among children under five in Uganda: A joint modeling analysis
Source: PLOS Glob Public Health. 2026 Oct 1;6(10):e0007387. doi: 10.1371/journal.pgph.0007387 (PMC13630190; doi:10.1371/journal.pgph.0007387)
Supplement: Supplementary file 6 [file pgph.0007387.s003.docx]

**S1 File. Maximum Simulated Likelihood Estimation for the Gaussian Copula Model**
Because the outcomes are binary, the joint likelihood under the Gaussian copula does not admit a closed-form expression and must be evaluated using simulation-based methods.

**Marginal transformation**
For each outcome $j$, the probit marginal implies

$$u_{ij}=\Phi(X_{ij}^{\top}\beta_{j}),$$

where $\Phi(\cdot)$ is the standard normal cumulative distribution function.

**Joint probability**
Let $(Z_{i1},Z_{i2})\sim N(0,\Sigma)$. The joint probability of observing $Y_{i}=(y_{i1},y_{i2})$ is

$$Pr(Y_{i})=\int_{a_{1}}^{b_{1}} \int_{a_{2}}^{b_{2}} \phi_{\Sigma}(z_{1},z_{2}) dz_{1}dz_{2},$$

where the integration limits $(a_{j},b_{j})$ depend on the observed binary outcomes and the transformed marginals.

**Simulated likelihood**
Maximum simulated likelihood approximates the joint probability by drawing $S$ samples

$$z_{i}^{(s)}=(z_{i1}^{(s)},z_{i2}^{(s)})\sim N(0,\Sigma),$$

and computing

$$\hat{L}_{i}=\frac{1}{S}\sum_{s=1}^{S} \mathbf{1}\{z_{i}^{(s)}\in B(y_{i})\},$$

where $B(y_{i})$ denotes the integration region corresponding to the observed outcome vector.

The simulated log-likelihood is then

$$logL(\beta,\Sigma)=\sum_{i=1}^{n} log\hat{L}_{i},$$

which is maximized numerically with respect to $\beta_{1},\beta_{2}$, and $\Sigma$.
