# Supplementary material for: Co-occurrence of anaemia and malaria among children under five in Uganda: A joint modeling analysis
Source: PLOS Glob Public Health. 2026 Oct 1;6(10):e0007387. doi: 10.1371/journal.pgph.0007387 (PMC13630190; doi:10.1371/journal.pgph.0007387)
Supplement: Supplementary file 7 [file pgph.0007387.s004.docx]

**S2 File. Computation of Average Marginal Effects**
Coefficients from probit models represent effects on a latent index and are not directly interpretable as changes in outcome probabilities. We therefore computed Average Marginal Effects (AMEs) to summarize covariate effects on the probability scale.

For individual $i$ and outcome $k$, the predicted probability is

$$Pr(Y_{ik}=1\mid X_{i})=\Phi(X_{i}^{\top}\beta_{k}).$$

For a covariate $x_{j}$, the individual-level marginal effect is calculated as the difference in predicted probabilities under two covariate values:

$$\Delta P_{ij}=Pr(Y_{ik}=1\mid X_{i}^{(j+1)})-Pr(Y_{ik}=1\mid X_{i}^{(j)}).$$

The AME is obtained by averaging these differences over all individuals:

$$\text{AME}_{j}=\frac{1}{N}\sum_{i=1}^{N} \Delta P_{ij}.$$

This quantity represents the population-average change in outcome probability associated with the covariate.
