# Supplementary material for: Co-occurrence of anaemia and malaria among children under five in Uganda: A joint modeling analysis
Source: PLOS Glob Public Health. 2026 Oct 1;6(10):e0007387. doi: 10.1371/journal.pgph.0007387 (PMC13630190; doi:10.1371/journal.pgph.0007387)
Supplement: Supplementary file 8 [file pgph.0007387.s005.docx]

**S3 File. Sensitivity Analyses and Robustness Checks**

**Generalized linear mixed models**
As a robustness check, we estimated outcome-specific generalized linear mixed models for anaemia and malaria using probit links. For child $i$ in household $h$ within survey cluster $c$, the model was specified as

$$\Phi^{-1}\{Pr(Y_{ij}=1\mid X_{ij},u_{c},v_{hc})\}=X_{ij}^{\top}\beta_{j}+u_{c}+v_{hc},$$

where $u_{c}$ and $v_{hc}$ represent cluster-level and household-within-cluster random intercepts, respectively.

**Covariates and random effects**
Fixed effects included child, maternal, household, and contextual characteristics. Random effects were specified at the primary sampling unit level, with a nested household-level random intercept in sensitivity specifications.

**Bootstrap and survey-weighted checks**
Inference robustness was assessed using a PSU-level, strata-preserving bootstrap. Degenerate resamples were excluded, and at least 300 valid replicates were retained. As an additional check, survey-weighted marginal probit models were estimated using replicate-weight designs, and parametric bootstrap confidence intervals were computed around the GLMM estimates.

**Diagnostic checks**
Multicollinearity among covariates was evaluated using variance inflation factors. No evidence of problematic multicollinearity was observed.
