# Supplementary material for: Co-occurrence of anaemia and malaria among children under five in Uganda: A joint modeling analysis
Source: PLOS Glob Public Health. 2026 Oct 1;6(10):e0007387. doi: 10.1371/journal.pgph.0007387 (PMC13630190; doi:10.1371/journal.pgph.0007387)
Supplement: Supplementary file 9 [file pgph.0007387.s006.docx]

**S4 File. Supplementary Results: Preliminary Evidence of Dependence Between Anaemia and Malaria**
As an initial empirical assessment of dependence between childhood anaemia and malaria, we examined the joint distribution of the two binary outcomes using log-linear models. These models were fitted to the cross-classified contingency table of anaemia and malaria status and provide a flexible framework for testing departures from independence without conditioning on covariates.
We compared a series of nested log-linear specifications, including mutual independence, joint independence, conditional independence, and homogeneous association models. Consistent with recent methodological work in similar epidemiological settings, the homogeneous association model provided the best fit to the observed data, closely approximating the saturated model, while simpler independence-based models exhibited substantial lack of fit.
The homogeneous log-linear model was specified as:

$$log(\mu_{ik})=\lambda+\lambda_{i}^{A}+\lambda_{k}^{M}+\lambda_{ik}^{AM},$$

where $\mu_{ik}$ denotes the expected cell count for anaemia status $i$ and malaria status $k$, and $\lambda_{ik}^{AM}$ represents the interaction term capturing dependence between the two conditions.
Estimation results are presented in Table A. The interaction parameter between anaemia and malaria was positive and statistically significant (estimate = 0.971, p < 0.01), corresponding to an odds ratio of 2.64. This indicates that the odds of anaemia were higher among malaria-positive children compared with malaria-negative children at the population level. The presence of a statistically significant interaction term supports the existence of outcome dependence and motivated the use of joint modeling approaches in the primary analysis.

**Table A. Parameter estimates from the homogeneous log-linear model for anaemia and malaria**

| Parameter | Estimate | Std. Error | z value | P-value |
| --- | --- | --- | --- | --- |
| Intercept | 7.845 | 0.019 | 402.06 | < 0.001 |
| Anaemia | −1.568 | 0.041 | −35.92 | < 0.001 |
| Malaria | −1.169 | 0.043 | −27.47 | < 0.001 |
| Anaemia × Malaria | 0.971 | 0.059 | 16.36 | < 0.001 |
