# Supplementary material for: Co-occurrence of anaemia and malaria among children under five in Uganda: A joint modeling analysis
Source: PLOS Glob Public Health. 2026 Oct 1;6(10):e0007387. doi: 10.1371/journal.pgph.0007387 (PMC13630190; doi:10.1371/journal.pgph.0007387)
Supplement: Supplementary file 10 [file pgph.0007387.s007.docx]

**S5 File. Supplementary Results: Simulation-Based Sensitivity Analyses and Model Validation**
To assess the robustness of the joint Gaussian copula framework, we conducted simulation-based sensitivity analyses designed to reflect key features of the empirical data. Synthetic datasets were generated to mirror observed marginal prevalences of anaemia and malaria, covariate distributions, and the estimated dependence structure between outcomes.
Binary outcomes were simulated using Bernoulli processes, with dependence imposed via a Gaussian copula parameterized using estimates from the empirical analysis. Covariates were sampled based on observed categorical proportions and continuous distributions. To reflect realistic survey conditions, stochastic noise and missingness were introduced, with missingness mechanisms partially dependent on selected covariates.
Simulations were conducted across varying sample sizes (1,000; 3,000; 5,000; and 7,000 observations) to evaluate the sensitivity of parameter estimates and predictive performance to data availability. For each simulated dataset, the joint copula model was re-estimated and evaluated using marginal effect recovery, confidence interval coverage, classification accuracy, and stability of the estimated dependence parameter.
Across simulation scenarios, the joint model consistently recovered the true marginal effects and dependence parameters with minimal bias. Performance metrics remained stable under increasing noise and partial missingness, and confidence interval coverage was close to nominal levels. These findings support the robustness of the joint modeling framework under conditions resembling those encountered in large-scale household surveys.
Additional validation metrics from the empirical analysis indicated good outcome-level discrimination, particularly for anaemia, with acceptable performance for malaria. Detailed numerical results from the simulations are available from the authors upon request.
