# Supplementary material for: Co-occurrence of anaemia and malaria among children under five in Uganda: A joint modeling analysis
Source: PLOS Glob Public Health. 2026 Oct 1;6(10):e0007387. doi: 10.1371/journal.pgph.0007387 (PMC13630190; doi:10.1371/journal.pgph.0007387)
Supplement: Supplementary file 11 [file pgph.0007387.s009.docx]

Rebuttal to Reviewer and Editor Comments

**Manuscript title:** Co-occurrence of Anaemia, Fever, and Malaria Among Children Under Five in Uganda: A Joint Modeling Analysis

**Manuscript ID:** PGPH-D-25-01028

**Dear Editor,**

We thank you and the reviewers for the thoughtful feedback. Below we respond point-by-point

# 1) “In the methodology, there is no mention of any stratification adopted.”

**Response.** We now explicitly describe the UMIS stratification and how it is handled analytically. The survey was stratified by region × urban/rural with an additional refugee stratum, and enumeration areas (EAs) served as primary sampling units (PSUs). Descriptive statistics were produced with a survey design object incorporating PSUs, strata, and sampling weights; standard errors account for the design.

# 2) “There is no mention about the sampling weights.”

**Response.** We now state the exact DHS/UMIS weight used and how it was applied. For the merged KR–PR analytic file, we use the child/person weight hv005 (scaled by 1,000,000) as case weights. All descriptive prevalence estimates are survey-weighted.

# 3) “Details about how much missing data, non-response rate, and how it was handled could be added.”

**Response.** We distinguish unit non-response (addressed through DHS/UMIS weighting) from item non-response. We added a sample-flow table and variable-wise missingness summary; exclusions due to missing outcomes/covariates are reported.

# 4) “It was not clear what methodology was adopted to select this sample out of the total dataset.”

**Response.** We added a dedicated subsection detailing inclusion/exclusion criteria and the order applied. Briefly: we began with all de facto children aged 0–59 months (PR), linked KR covariates, retained records with valid malaria and haemoglobin results, and then applied complete-case requirements for modelling covariates. The final analytic sample comprised 5,755 children (80.8% of 7,124 eligible).

# 5) “How was fever-related data collected? Self-reported fever and/or assessment using any valid instrument (thermometer)?”

**Response.** In 2018–19 UMIS, fever among under-fives was caregiver-reported for the prior two weeks (yes/no). No thermometer-based temperatures were collected in the biomarker module; biomarkers were limited to haemoglobin and malaria testing (RDT/microscopy). Given fever’s self-reported nature and the temporal mismatch with point-in-time biomarkers, we dropped fever a priori as an analytical outcome for the joint model. We now state this explicitly and retain fever, where relevant, only for descriptive context.

# 6) “It is preferred to mention the validation of the model in terms of either Loglikelihood ratio, Pseudo R², AIC/BIC, or any other estimate.”

**Response.** We report a comprehensive set of fit and performance metrics for the joint bivariate probit (Gaussian copula) and, for comparison, separate survey-weighted probits: Joint model (weighted): log-likelihood = −0.0043; AIC = 118.01; BIC = 510.82. Copula correlation ρ = 0.191 with PSU-level, strata-preserving bootstrap 95% CI [0.114, 0.263]. Outcome-level performance (from joint model): Tjur’s R² = 0.606 (anaemia) and 0.232 (malaria); AUC = 0.943 and 0.798; Brier scores = 0.090 and 0.159. Separate survey-weighted probits: McFadden’s pseudo-R² = 0.599 (anaemia) and 0.206 (malaria). A gradient test yielded p = 0.996; because this test is not design-consistent, we prioritize the bootstrap CI for inference on ρ.

# 7) “Elaboration on GLMM will be preferred, which variables were considered to have a fixed effect, and the random effect can be mentioned.”

**Response.** We added a sensitivity specification using GLMMs (binomial-probit link). Fixed effects include all child, maternal, and household predictors used in the main model (age group, sex, wealth index, education, antenatal visits, maternal anaemia, maternal age group, residence, toilet and water facility, region, number of children ever born, religion). We include a random intercept at the survey cluster (PSU, hv021), and in sensitivity analyses a nested household random intercept (HHID) within PSU when applicable. Models use probability weights (hv005/1,000,000); uncertainty is summarized with a PSU-level, strata-preserving bootstrap. Fixed-effect inferences are qualitatively consistent with the copula model.

# 8) “Confidence Interval for Prevalence Estimates can be provided.”

**Response.** We now provide CIs for all unweighted prevalence percentages of outcome combinations (binomial CIs): Anaemia only: 43.0% (95% CI: 41.3–44.7); Malaria only: 29.6% (95% CI: 28.1–31.2); Both malaria and anaemia: 27.4% (95% CI: 25.9–28.9).

# 9) “Rationale should be a part of the introduction, not the results.”

**Response.** We agree that the conceptual rationale belongs in the Introduction and have retained a concise statement there to motivate joint modelling. We also keep a brief subsection in the Results, explicitly framed as empirical evidence for outcome dependence and model choice—i.e., diagnostics derived from our data (co-occurrence rates and copula correlation). Presenting these diagnostics alongside the prevalence findings helps readers see why the joint model is warranted. This is a common practice when the diagnostics are themselves results.

# 10) “Divergent results between the separate model and the combined model could have been discussed with detailed literature support.”

**Response.** We expanded the Discussion to explain why inference can differ between separate probits and a joint (Gaussian-copula / multivariate probit) specification. When outcomes share latent determinants, separate models ignore cross-equation error correlation, which can reduce efficiency and shift marginal effects once dependence is modeled jointly. Copula-based multivariate probit models estimate this residual dependence and borrow strength across equations, typically yielding more stable and interpretable effects than separate fits. We also clarify how GEE differs (population-averaged means without a joint outcome distribution). Citations have been added accordingly.

# 11) “Correlation can be expressed as low/moderate or high (based on any of the available classification systems), instead of reporting as statistically significant.”

**Response.** We revised the Results to describe the magnitude of dependence instead of significance testing language. Using widely used benchmarks, ρ = 0.191 (95% CI: 0.124–0.244) is characterized as a small/weak positive correlation on the latent scale of the Gaussian copula.

Sincerely,

**Grace Kakaire**
on behalf of all authors
