# Supplementary material for: Co-occurrence of anaemia and malaria among children under five in Uganda: A joint modeling analysis
Source: PLOS Glob Public Health. 2026 Oct 1;6(10):e0007387. doi: 10.1371/journal.pgph.0007387 (PMC13630190; doi:10.1371/journal.pgph.0007387)
Supplement: Supplementary file 12 [file pgph.0007387.s011.docx]

**Rebuttal to Reviewer and Editor Comments**

**Manuscript title:** Co-occurrence of Anaemia, Fever, and Malaria Among Children Under Five in Uganda: A Joint Modeling Analysis

**Manuscript ID:** PGPH-D-25-01028

**Dear Editor,**

We thank you and the reviewers for the thoughtful feedback. Below we respond point-by-point

**Reviewer Comment:**
*The Introduction section on the body of manuscript needs revision so that it will cover the nature of the core problem, the known aspect of the problem, the unknown aspect of the problem, and the expectations from the current study.*

**Response:**
We appreciate this important observation. In response, we have substantially revised the Introduction to clearly and explicitly address each of the elements highlighted by the reviewer.

Specifically, the revised Introduction now:

1. **Clearly defines the nature of the core problem** by framing childhood anaemia and malaria in sub-Saharan Africa as a major public health challenge characterized by high prevalence, shared risk factors, and frequent co-occurrence among children under five.
2. **Summarizes what is currently known** by synthesizing existing evidence on the epidemiology of anaemia and malaria, their shared biological and socioeconomic determinants, and the dominant use of univariate analytical approaches in previous studies.
3. **Identifies the key knowledge gap** by explicitly highlighting the limited empirical understanding of the dependence structure between anaemia and malaria at the individual level, particularly the lack of studies that quantify latent correlations between these conditions after accounting for observed covariates.
4. **Clearly states the expectations and contribution of the current study** by emphasizing the use of a joint modeling framework to estimate both marginal effects and residual dependence between anaemia and malaria, thereby advancing understanding of childhood multimorbidity and informing integrated health interventions.

These revisions have strengthened the conceptual framing of the study and clarified how the current work advances existing knowledge beyond disease-specific analyses. The Introduction has been reorganized and expanded accordingly (see revised Introduction section).

**Reviewer Comment:**
*In page 7 and line 130–131: “Children aged 0–59 months were tested for both anaemia and malaria with the parents' or guardians’ consent.” Is it possible that children aged 0–59 months were tested?*

**Response:**
We thank the reviewer for highlighting this important clarification. In the UMIS, malaria testing (RDT and microscopy) is conducted among children aged **0–59 months**, whereas anaemia testing is performed among children aged **6–59 months**. The statement in the manuscript has been revised to accurately reflect these eligibility criteria. The corrected text now reads:

*“Children aged 0–59 months were eligible for malaria testing, while anaemia testing was conducted among children aged 6–59 months, with parental or guardian consent.”*

This revision ensures consistency with DHS/UMIS biomarker protocols.

**Reviewer comment:**
Please include as a Table the shows the multicollinearity assumption with its mean of VIF test of the variables in the supplementary materials.

**Response:**
We thank the reviewer for this suggestion. In response, we formally assessed multicollinearity among all explanatory variables included in the joint model using Variance Inflation Factors (VIFs). VIFs were computed from an auxiliary linear regression including the full set of covariates used in the analysis, which is standard practice for assessing collinearity.

The results are now presented in **Supplementary Table S1**, which reports individual VIF values for each covariate as well as the mean VIF. All VIFs were below conventional thresholds, and the mean VIF indicated no evidence of problematic multicollinearity. These results confirm that the multicollinearity assumption is satisfied for the models estimated in this study.

**Reviewer comment:**

What type of statistical software do you use (i.e., R, STATA, SAS...) in your analysis, and please upload as supplementary material the codes/commands of the statistical software?

**Response:**

We thank the reviewer for this request. All statistical analyses in this study were conducted using **R statistical software (version 4.4.2)**. To improve transparency and reproducibility, we have now uploaded the full set of R scripts used for data preparation, model estimation (separate probit models and joint Gaussian copula models), and post-estimation analyses as **Supplementary Materials (S2 File)**. The Methods section has been updated to explicitly state the statistical software and version used in the analysis.

**Reviewer comment:**

Please use the latest reference rather than old reference! Like reference 2, 3, 21 etc.

**Response:**

We thank the reviewer for this important suggestion. In response, we have carefully reviewed the reference list and updated the manuscript to incorporate **more recent and relevant literature**, particularly for references originally cited as 2, 3, and 21. Where appropriate, older references have been replaced with or supplemented by **up-to-date studies and reviews** that reflect current evidence and methodological advances in childhood morbidity, malaria, anaemia, and joint modeling approaches. These updates have strengthened the contextual relevance of the Introduction and ensured that the study is grounded in the most recent literature.

**Reviewer comment:**

Please approximate the results of tables of the analysis into 3 digits in the Results section (e.g., 0.002, 1.12, etc.).

**Response:**

We thank the reviewer for this helpful suggestion. In response, we have revised the Results section and all corresponding tables to ensure that numerical estimates (including coefficients, marginal effects, standard errors, and p-values) are consistently reported to **three decimal places**. This formatting change has been applied throughout the manuscript to improve clarity, readability, and consistency with journal standards.

Sincerely,

**Grace Kakaire**
on behalf of all authors
