# Supplementary material for: Co-occurrence of anaemia and malaria among children under five in Uganda: A joint modeling analysis
Source: PLOS Glob Public Health. 2026 Oct 1;6(10):e0007387. doi: 10.1371/journal.pgph.0007387 (PMC13630190; doi:10.1371/journal.pgph.0007387)
Supplement: Supplementary file 13 [file pgph.0007387.s013.docx]

## :

## Reviewer Comments from Dr Adamu Gelaw

## Manuscript ID: PGPH-D-25-01028R2

# Title:

### Co-occurrence of Anemia & malaria Among Children under Five in Uganda-A joint Modeling analysis

A. **Research significance and contributions**

The manuscript covers an important public health issue in Sub-Saharan Africa where malaria and anemia co-occurrence is prevalent. Above and all the use of a joint Gaussian Copula modeling framework is innovative, and adds methodological rigor compared to other traditional univariate approaches. In the analysis findings that revealed the 27% co-occurrence, copula correlation P =0.19 provided valuable evidence that will be critical for an integrated child health interventions.

However, as a general minor comments: this great contribution could be strengthened by more explicitly contrasting results with the traditional univariate studies, and situating findings within the broader syndemic framework

B. **Abstract**

It is well written addressing critical and important areas, my only comment is to make it more concise by removing methodological details: Gaussian copula framework with probit margins, but focusing on findings and implications.

C. **Methodology**

| S.No | Strengths | Concerns |
| --- | --- | --- |
| 1 | Data source description is very clear | Survey weights associated with the exclusion of DHS complex survey weights (as a limitation), lacking detailed discussions on potential biases |
| 2 | Highly transparent sample selection & biometric testing | There is no clear justification, why Gaussian copula selected out of other alternatives: why Clayton, Frank, and /or Gumbel were not tested |
| 3 | Use of probit margins within the copula framework appropriately | Not able to see the interpretation of correlation (P=0.19), though found significant, its clinical or epidemiological relevance |

D. Results and Interpretations

The result session is presented clearly with appropriate use of marginal effect. The only comment and areas to fine-tune is

- The striking association of Maternal anemia with the child malaria risk in the joint model needs deeper exploration: biological, social mechanisms
- Try to concretely articulate policy implications: For instance how integrated screening and/or treatment programs can be designed differently.
- Some limitation that are already acknowledged should be expanded: (Example exclusion of survey weights in modeling, restrictions to age group 6-59, potential misclassification bias in Malaria diagnosis-(RDT/Microscopic), and potential generalizability beyond Uganda)

E. Other Minor comments

- Check for consistency (anaemia/anemia)
- Check and reformat all references
- Review the repetition of ideas over section e.g. Background/discussion

## Overall Recommendation

**Minor revision:** The manuscript is promising in its novelty, methodological approaches. And, it is commendable analysis.

- Nevertheless, its representativeness poses a concern because of the exclusion of survey weights in the copula model.
- The authors should provide clear clarifications on the potential impact of its limitation, and
- If need be they should conduct sensitivity analysis
- Conduct deeper discussion on the reversal of maternal anemia associated with child malaria risk, though said to be corrected.
