# Supplementary material for: Co-occurrence of anaemia and malaria among children under five in Uganda: A joint modeling analysis
Source: PLOS Glob Public Health. 2026 Oct 1;6(10):e0007387. doi: 10.1371/journal.pgph.0007387 (PMC13630190; doi:10.1371/journal.pgph.0007387)
Supplement: Supplementary file 14 [file pgph.0007387.s014.docx]

The manuscript titled *Co-occurrence of Anaemia and Malaria Among Children Under Five in Uganda: A Joint Modeling Analysis" (PGPH-D-25-01028R2)* presents a significant effort in analyzing the anemia and malaria as a joint model in Uganda. The use of a Gaussian copula–based bivariate probit model is methodologically sophisticated and potentially impactful for understanding childhood comorbidities. However, several major concerns should be addressed to improve clarity, epidemiological validity, and alignment with existing data. The following comments are intended to strengthen the manuscript’s contributions.

The paper’s reported prevalence rates for anemia, malaria, and their co-occurrence appear inconsistent with well-documented regional data. It states that 27% of children under five had both anemia and malaria, with 43% anemic-only and 30% malaria-only, implying ~70% overall anemia prevalence and ~57% malaria prevalence. While a 70% anemia rate is plausible, the 27% co-prevalence seems low compared to expectations from endemic settings. Further, if anemia and malaria were independent, ~40% co-prevalence would be expected (0.7 × 0.57).

Specifically:

If Table 2 truly uses the full sample as the denominator, then

P(anemia | malaria) = 27.4 / (27.4 + 29.6) ≈ 48%,

which is far below the ~70–80% range typically reported for malaria-positive children in high-burden settings.

And P(malaria | anemia) = 27.4 / (27.4 + 43.0) ≈ 39%.

WHO data show anemia is extremely widespread in high-malaria regions (≥60% of under-5 children) and that around 79% of children with malaria infection are anemic in high-burden African countries. ^1^ In Uganda specifically, national surveys have historically found >60% anemia prevalence in under-fives (e.g. 72% in 2006), and over half of children tested positive for malaria in the 2009 survey. Moreover, a recent Ugandan study in an eastern region reported 67.4% anemia prevalence in under-5. ^2, 3^ Crucially, in malarial areas the vast majority of malaria-positive children are typically anemic; for instance, a Ghanaian study found nearly 70% of parasitemic children were concurrently anemic. ^4^ Given this context, a co-infection rate of only 27% is surprisingly low. It suggests that many children in the survey who had malaria did not present with anemia, and vice-versa, to a degree that conflicts with the strong biological link between malaria and hemolytic anemia in endemic zones. This discrepancy raises concerns about potential underestimation or misclassification of the data. The authors should address this gap explicitly. Is it because a large proportion of anemia cases were due to other causes (nutrition, helminths, etc.)? Or did the survey timing/method miss some malaria anemia overlaps? The manuscript would be strengthened by comparing its findings to WHO/DHS figures and explaining why the co-prevalence is lower than expected. If the results are indeed accurate, a discussion is needed on how this specific studies’ context might differ (e.g. improved malaria control or nutrition reducing co-morbidity or geography).

Further, if this is secondary to MIS methodology this should be explicity mentioned, including in limitations, specifically the diagnostic method (RDT vs. Microsopy), age range as well as selective nature of the participation and the cross sectionality.

From a statistical standpoint, the use of a joint Gaussian copula–based bivariate probit model is an innovative approach to assess dependency between outcomes. However, there are concerns about how the complex survey design was handled in the modeling, which could affect prevalence estimates and inferences. Specifically, the authors indicate that DHS/MIS sampling weights were used for descriptive analyses, but it appears the model itself did not incorporate survey weights or clustering. (My understanding is that a PSU-level bootstrap in sensitivity analyses was attempted, implying the main model was unweighted.) If true, this is problematic because ignoring weights can bias the estimated prevalences and associations. The MIS oversamples certain regions and uses cluster sampling; not accounting for this may “flatten” extremes, underestimating true national anemia in high-risk strata and diluting malaria–anemia covariance. As a reviewer, I recommend the authors explicitly clarify how weights and clustering were handled in the joint model and consider using survey-weighted techniques (or at least robust sandwich corrections) for accuracy. If the copula approach cannot easily accommodate weights, this should be stated as a limitation, and the consistency of results with a weighted analysis should be shown (the manuscript hints that results were similar, but providing those numbers would increase confidence).

Concurrently, with the issue raised initially, the copula correlation (ρ ≈ 0.19) indicated a modest positive dependency between anemia and malaria. Given the strong biological link, one expects a higher correlation. The relatively low ρ could be a result of the aforementioned measurement issues and the inclusion of many covariates (some shared risk factors). In adjusting for covariates, the model may have “explained away” some covariance (e.g. common determinants like age, nutrition, and geography). It might reflect that older children had more malaria but less anemia (and younger vice versa), or other confounding patterns that the copula tries to account for. The authors should ensure the interpretation of ρ = 0.19 is clear, i.e. it’s a latent correlation after controlling for covariates. It would be constructive for the authors to discuss why this adjusted correlation isn’t higher, touching on the data limitations and the presence of many anemia cases unrelated to malaria (e.g. nutritional anemia) diluting the malaria–anemia link. Alongside, Uganda has a high burden of Sickle Cell Disease (SCD) and Alpha-thalassemia. These genetic traits are arguably the most important "unobserved" factors that would link malaria and anemia, yet they are entirely absent from the analysis. Since the MIS likely did not test for SCD, this must be added as a major limitation. Common hemoglobinopathies (e.g., sickle cell disease/trait and α-thalassemia) may affect hemoglobin levels and malaria outcomes, and are not captured in MIS; this should be acknowledged as an unmeasured factor contributing to the estimated latent dependence.

In the same sense and in line with above comments, please ensure that the prevalence estimates are compared to external benchmarks beyond introduction but in the setting of discussion and appropriately contextualized.

Specific Comments:

- In Table 2, the three listed categories (malaria only 29.6%, anemia only 43.0%, both 27.4%) sum to ~100%. This implies essentially 0% of children had neither malaria nor anemia, which is counter-intuitive. Presumably a small fraction of the sample had no anemia and tested negative for malaria. The authors should clarify this. If the percentages were calculated among children who had at least one of the illnesses, that should be stated. Otherwise, if truly >99% of under-5s were either anemic or malaria-infected, that is an astonishing finding that warrants emphasis (and likely reflects the high endemicity). It would be clearer to report the percentage with neither condition as well, or note it in text, for completeness.
- Please confirm the hemoglobin cutoff used for anemia (<11 g/dL, presumably per WHO). Also note if values were altitude-adjusted. The Methods mention hemoglobin measurement but I did not see a statement of the threshold. Adding “(Hb <11.0 g/dL, per WHO definition of anemia in U5 children)” on first mention would be helpful for readers.
- The Results briefly mention an interesting finding that maternal anemia had a reversed association with child malaria in the joint model (versus separate models). This is an important epidemiological insight. In the Discussion, consider elaborating on plausible explanations (e.g. maternal anemia might be a proxy for other unmeasured factors that, once accounting for child’s anemia, inversely relate to malaria susceptibility, perhaps care-seeking or nutritional status differences). Currently it’s mentioned in passing; a sentence or two interpreting that result would add value. The same goes for any other covariate effects that differed in joint vs. independent models.
- A minor suggestion is to comment on how generalizable these findings are across seasons and regions. Uganda has known regional heterogeneity in malaria and anemia (e.g., East-Central and northern regions historically have higher burdens than southwestern). Since your model adjusted for region, it would be worth noting if the dependency between malaria and anemia differed by region or was fairly consistent. If certain regions (perhaps those with holoendemic malaria) had especially high co-morbidity, that could be mentioned.
- The paper concludes by emphasizing the importance of joint disease modeling for policy. To make this more actionable, consider mentioning specific interventions. For example, since malaria contributes to anemia, integrated approaches like combining anti-malarial measures with anemia control (iron supplementation, nutritional programs) are indicated. WHO recommends treating malaria in anemic children and vice versa; perhaps suggest that screening anemic children for malaria (and underlying causes) could be beneficial in high-risk areas. Current text is method-focused; adding a public health perspective would round it out.
- While the writing is clear and well structured, a few editorial points, ensure consistent terminology (sometimes “anaemia” vs “anemia”), and check that all acronyms are defined.
- The methods mention that "Thin smears... were read to determine the species of Plasmodium parasite". However, the results treat malaria as a binary positive/negative. Was P. falciparum (the primary driver of severe hemolysis) dominant? Mentioning the species breakdown would strengthen the biological plausibility of the anemia link.
- The discussion states the joint model establishes that illnesses "don't arise independently". Medically, Malaria is a direct cause of anemia, while anemia/nutritional status may also modify susceptibility and care-seeking; cross-sectional data cannot establish directionality. The language should be softened to reflect that the "dependence" is likely unidirectional.

1.	Organization WH. World malaria report 2025: Addressing the threat of antimalarial drug resistance. World Health Organization; 2025.

2.	Komakech JJ, Nsubuga EJ, Graves JM, et al. Determinants of anemia among children under five in Eastern Uganda: a community-based cross-sectional study. Frontiers in Public Health. 2025; 13doi:10.3389/fpubh.2025.1618395

3.	Menon MP, Yoon SS, Uganda Malaria Indicator Survey Technical Working G. Prevalence and Factors Associated with Anemia Among Children Under 5 Years of Age--Uganda, 2009. Am J Trop Med Hyg. Sep 2015; 93(3):521-526. doi:10.4269/ajtmh.15-0102

4.	Dao F, Djonor SK, Ayin CT-M, et al. Burden of malaria in children under five and caregivers’ health-seeking behaviour for malaria-related symptoms in artisanal mining communities in Ghana. Parasites & Vectors. 2021; 14(1)doi:10.1186/s13071-021-04919-8
