# Supplementary material for: Co-occurrence of anaemia and malaria among children under five in Uganda: A joint modeling analysis
Source: PLOS Glob Public Health. 2026 Oct 1;6(10):e0007387. doi: 10.1371/journal.pgph.0007387 (PMC13630190; doi:10.1371/journal.pgph.0007387)
Supplement: Supplementary file 15 [file pgph.0007387.s015.docx]

**Rebuttal to Reviewer and Editor Comments**

**Manuscript title:** Co-occurrence of Anaemia, Fever, and Malaria Among Children Under Five in Uganda: A Joint Modeling Analysis

**Manuscript ID:** PGPH-D-25-01028

Dear Editor,

We thank you and the reviewers for the thoughtful feedback. Below, we address the comments of **Reviewer 3, Reviewer 4, Reviewer 6, and Reviewer 7** point by point. All changes are reflected in the revised manuscript, with additional material provided in the Supplementary Files where appropriate.

**Editorial comment**

Please review your reference list to ensure that it is complete and correct. If you have cited papers that have been retracted, please include the rationale for doing so in the manuscript text, or remove these references and replace them with relevant current references. Any changes to the reference list should be mentioned in the rebuttal letter that accompanies your revised manuscript. If you need to cite a retracted article, indicate the article’s retracted status in the References list and also include a citation and full reference for the retraction notice.

**Response**

We carefully reviewed the entire reference list to ensure accuracy and currency, including screening for retracted publications. No cited articles were found to be retracted. We corrected incomplete bibliographic information, standardized journal names and formatting, removed a duplicate citation of the Uganda Demographic and Health Survey. These changes have been reflected in the revised manuscript and reference list.

**Reviewers 4, 6 & 7**

**Reviewer comment**

Internal consistency and interpretation of prevalence/comorbidity (Table 2):
Table 2 reports “anemia only” 43.0%, “malaria only” 29.6%, and “both” 27.4%, summing to ~100%. This implies essentially 0% of children had neither anemia nor malaria, which is highly implausible and requires clarification of the denominator and definitions. Please provide the full 2×2 table including the “neither” category and clearly state the denominator used. As written, these figures also imply P(anemia | malaria) ≈ 48%, which appears unexpectedly low for malaria-endemic settings.

**Response**

We thank the reviewer for identifying an important issue in the presentation of Table 2. The earlier percentages were inadvertently reported conditional on children with at least one condition, which obscured the “neither” category. We have revised Table 2 to present the full 2×2 distribution, including children with neither anaemia nor malaria, and we now clearly state the denominator (children with complete biomarker data for both outcomes). In the revised Table 2, 49.481% of children had neither condition, 21.720% had anaemia only, 14.977% had malaria only, and 13.822% had both conditions. We also added conditional prevalences to aid interpretation: P(anaemia | malaria) = 47.994% and P(malaria | anaemia) = 38.889%.

Using the corrected table, the conditional prevalence P(anaemia | malaria) = 47.99%, which we now report explicitly in the Results. We note that this value reflects point-prevalence malaria status and the multifactorial causes of anaemia and is consistent with prior MIS-based estimates in malaria-endemic settings.

**Reviewer comment**

External validity: co-occurrence appears lower than expected given endemic biology and benchmarks:
While overall anemia prevalence around ~70% can be plausible in high-burden settings, the implied overlap (e.g., anemia among malaria-positive children) appears low relative to published data from high-burden African settings and Uganda. The Discussion should explicitly compare the study’s conditional prevalences (e.g., P(anemia|malaria), and P(malaria|anemia)) to external benchmarks and explain potential reasons for divergence (case definitions, diagnostic modality, temporal mismatch between infection and anemia, geography, improvements in malaria control, or competing causes of anemia such as nutrition/helminths).

**Response**

We thank the reviewer for raising the issue of external validity regarding co-occurrence magnitudes. We have revised the Discussion to explicitly benchmark our conditional prevalences (P(anaemia | malaria) = 47.994%; P(malaria | anaemia) = 38.889%) against published evidence from Uganda and other high-burden African settings. For instance, earlier Uganda MIS-based analyses reported higher overlap (e.g., 77.0% anaemia among malaria-positive children and 64.9% malaria positivity among anaemic children), which we now cite and discuss. We also added plausible explanations for divergence, including differences in case definitions and diagnostic modality (microscopy vs RDT and potential RDT false-positives), temporal mismatch between point-prevalence malaria infection and longer-duration anaemia, geographic heterogeneity, changing transmission intensity and malaria control progress, and competing non-malarial causes of anaemia such as nutrition and helminths.

**Reviewer Comment**

MIS measurement constraints should be stated clearly as major interpretive limitations, including haemoglobin measured only in children aged 6–59 months, malaria defined using microscopy vs. RDT, cross-sectional timing, and potential selection bias if severely ill children were less likely to be captured in household surveys.

**Response**

Response: We thank the reviewer for highlighting the importance of clearly articulating MIS measurement constraints. We have revised the Methods and Discussion (Limitations) sections to explicitly acknowledge key limitations inherent to secondary analysis of MIS data. Specifically, we now clarify that haemoglobin was measured only among children aged 6–59 months, that malaria infection was defined using microscopy-confirmed parasitaemia, which reflects point-prevalence infection, and that anaemia may persist after malaria clearance in a cross-sectional design. We also discuss the potential for selection bias if severely ill or hospitalized children were underrepresented in household surveys. These additions provide essential context for interpreting prevalence and co-occurrence estimates and strengthen the external validity and transparency of the study.

**Reviewer Comment**

Complex survey design handling in the joint model: The manuscript notes that descriptive estimates account for MIS survey design, but the joint copula model appears unweighted / not fully design-based. Please clearly state how weights, clustering, and stratification were handled in the joint model, and provide sensitivity analyses (or robust SEs / alternative approaches) to demonstrate that inferences are not materially biased by ignoring complex survey design. If the modeling framework cannot accommodate survey weights, this should be highlighted as a limitation and key results should be shown alongside survey-weighted benchmarks.

**Response**

We thank the reviewer for this important and constructive comment. We have revised the manuscript to clearly distinguish between design-based and model-based components of the analysis and to transparently describe how the MIS complex survey design was handled.

Specifically, all descriptive statistics and prevalence estimates now explicitly account for DHS sampling weights, clustering at the primary sampling unit (PSU) level, and stratification to ensure national representativeness. For the joint Gaussian copula regression, we clarify that the current implementation of the GJRM framework does not natively support survey-weighted likelihood estimation or full design-based inference. Consequently, the joint model was estimated without direct application of survey weights, and this limitation is now explicitly acknowledged in both the Methods and the Discussion sections.

To assess the potential impact of this limitation, we conducted a series of sensitivity analyses. These included (i) survey-weighted marginal probit regressions as design-based benchmarks, (ii) generalized linear mixed models with PSU-level (and household-level) random intercepts to account for clustering, and (iii) bootstrap procedures preserving the PSU structure. Across these alternative specifications, the direction, magnitude, and statistical significance of key covariate effects, as well as the positive residual dependence between anaemia and malaria, were consistent with the joint copula model results.

These revisions demonstrate that our main conclusions are robust to alternative treatments of the complex survey design, while transparently acknowledging current methodological constraints. Corresponding text has been added to the Methods and Limitations sections, and relevant results are referenced in the Supplementary Materials.

**Reviewer Comment**

Interpretation of the dependence parameter (ρ) and unmeasured determinants: The estimated copula correlation (ρ ≈ 0.19) reflects latent residual dependence after covariate adjustment and should be interpreted accordingly. The Discussion should address why ρ is modest despite strong malaria→anemia pathophysiology (possible dilution by non-malarial anemia, age-pattern differences, measurement limitations). Additionally, common hemoglobinopathies in Uganda (e.g., sickle cell disease/trait, α-thalassemia) can influence hemoglobin and malaria outcomes and are not captured in MIS; this should be acknowledged as an important unmeasured factor potentially contributing to the latent dependence.

**Response**

We thank the reviewer for this insightful comment and fully agree that the dependence parameter requires careful interpretation. We have revised the Discussion to explicitly clarify that the estimated copula correlation reflects *residual latent dependence* between anaemia and malaria after adjustment for observed covariates, rather than a direct causal effect.

The revised text explains why the estimated correlation is modest despite strong biological links, highlighting (i) the heterogeneous, multi-cause etiology of childhood anaemia, (ii) age-specific and temporal mismatches between malaria infection and anaemia persistence in cross-sectional data, and (iii) measurement constraints related to microscopy-based malaria diagnosis and age-restricted haemoglobin testing in the MIS. We also explicitly acknowledge the absence of key biological determinants—particularly hemoglobinopathies such as sickle cell trait/disease and α-thalassemia, which are prevalent in Uganda—as an important source of unmeasured confounding that may attenuate the estimated dependence.

These additions strengthen the biological plausibility and interpretive clarity of the findings and are now incorporated into the Discussion and Limitations sections.

**Reviewer Comment**

The manuscript occasionally uses language that may imply bidirectional causality between malaria and anaemia (e.g., statements suggesting that the illnesses “do not arise independently”). While malaria is a known biological cause of anaemia, cross-sectional survey data cannot establish causal directionality, and anaemia or nutritional status may also modify malaria susceptibility or care-seeking. The Discussion should emphasize association and residual dependence rather than causal inference.

**Response**

We thank the reviewer for this important clarification. We agree that the cross-sectional design of the Malaria Indicator Survey does not permit causal inference or temporal ordering between malaria and anaemia.

In response, we have carefully revised the manuscript to soften causal language throughout the Discussion and Results interpretation, replacing it with terminology that emphasizes association, co-occurrence, and residual dependence. Specifically, statements implying that the illnesses “do not arise independently” have been revised to indicate statistically significant residual dependence after covariate adjustment, as captured by the copula correlation parameter.

We have also added an explicit clarification in the Discussion noting that, although malaria is a known biological contributor to anaemia, the present analysis cannot establish directionality, bidirectional causation, or temporal sequencing. The estimated copula correlation (ρ) is therefore interpreted as reflecting latent association due to shared or unmeasured determinants, rather than causal effects.

**Reviewer Comment**

Please confirm the hemoglobin cutoff used to define anaemia (e.g., Hb <11 g/dL for children aged 6–59 months) and whether hemoglobin values were adjusted for altitude. Consider reporting anaemia severity categories (mild, moderate, severe) if available, since malaria-associated anaemia is often more severe and clinically meaningful.

**Response**

We thank the reviewer for this important methodological clarification.

We confirm that childhood anaemia was defined according to WHO and DHS/MIS guidelines, using a hemoglobin cutoff of Hb <11.0 g/dL for children aged 6–59 months. Hemoglobin measurements were obtained using a portable HemoCue analyzer, and altitude-adjusted hemoglobin values provided in the MIS dataset were used in all analyses. We have now explicitly stated both the cutoff and the use of altitude-adjusted hemoglobin values in the *Data Collection and Measurements* subsection of the Methods.

Regarding anaemia severity, the MIS dataset includes categorical classifications of anaemia severity (mild, moderate, severe). However, our primary objective in this study was to model the binary co-occurrence and residual dependence between malaria and anaemia using a joint copula framework. Stratifying the outcome by severity would substantially reduce cell sizes—particularly for severe anaemia—thereby limiting statistical power and complicating joint model estimation.

**Reviewer comment**
The Methods note species determination from thin smears, but results treat malaria as binary. If available, report species distribution (especially P. falciparum) and comment on biological plausibility. The reversal/attenuation of the maternal anemia association under joint modeling is interesting; consider adding a short mechanistic interpretation.

**Response**
We thank the reviewer for this insightful comment. We have clarified in the Methods and Results that microscopy-positive malaria infections were overwhelmingly due to *Plasmodium falciparum*, consistent with Uganda’s epidemiology, and that malaria was therefore modeled as a binary outcome to reflect the dominant, clinically relevant species while ensuring stable estimation. We have also added a brief discussion highlighting the biological plausibility of the malaria–anaemia association given *P. falciparum* pathophysiology.

**Comment**
Please comment briefly on generalizability across regions and seasons, given Uganda’s geographic heterogeneity. Also ensure consistent spelling, fix typos (e.g., microscopy), and define acronyms at first use.

**Response:**
We thank the reviewer/editor for these helpful suggestions. We have added a brief paragraph in the Discussion addressing the generalizability of our findings across Uganda’s regions and seasons, noting substantial geographic heterogeneity in malaria transmission and anaemia burden, as well as the peak-season timing of the MIS. We clarify that while national inference is supported, the magnitude of co-occurrence and residual dependence may vary by region and season.

In addition, we conducted a full editorial review of the manuscript to ensure consistent spelling (“anaemia” used throughout), corrected typographical errors (including microscopy), and defined all acronyms at first use in accordance with journal guidelines.

**Reviewer Comment**

There is no clear justification for selecting the Gaussian copula over alternative copula families such as Clayton, Frank, or Gumbel. The manuscript should explain why these alternatives were not tested or adopted.

**Response**

We thank the reviewer for this insightful comment. We have added a brief justification in the Methods section explaining our choice of the Gaussian copula. Specifically, the Gaussian copula was selected because it captures moderate, symmetric residual dependence between binary outcomes, which is appropriate for anaemia and malaria measured as point prevalence. There was no strong theoretical or empirical evidence of asymmetric tail dependence that would motivate Clayton or Gumbel copulas, while Frank offers limited epidemiological interpretability in this context. Given the binary nature of the outcomes and the focus on latent correlation rather than tail behavior, the Gaussian copula provides a parsimonious and stable specification consistent with the multivariate probit framework.

**Reviewer Comment**

The Results section is presented clearly with appropriate use of marginal effects. However, the following areas require further refinement:
(i) the strong association between maternal anaemia and child malaria risk observed in the joint model requires deeper exploration of potential biological and social mechanisms;
(ii) policy implications should be articulated more concretely, particularly regarding how integrated screening or treatment programs could be designed; and
(iii) some limitations already acknowledged should be expanded, including the exclusion of survey weights in joint modeling, restriction of the joint analysis to children aged 6–59 months, potential malaria misclassification (RDT/microscopy), and generalizability beyond Uganda.

**Response**

We thank the reviewer for these constructive suggestions and have revised the manuscript accordingly.

First, we expanded the Discussion to provide a clearer interpretation of the association between maternal anaemia and child malaria risk observed in the joint model. We now discuss plausible biological pathways (e.g., shared nutritional deficiencies, immune vulnerability, and maternal–child health linkages) as well as social mechanisms, including care-seeking behavior, household food insecurity, and correlated exposure to infection. We emphasize that the attenuation and sign reversal observed under joint modeling likely reflect correction for unobserved shared risk rather than a direct causal effect.

Second, we strengthened the policy implications by explicitly describing how the findings support integrated child–maternal health strategies, such as combined malaria testing and anaemia screening during routine child health visits, antenatal and postnatal follow-up that jointly addresses maternal anaemia and child infection risk, and coordinated nutrition–malaria control interventions in high-burden regions.

Third, we expanded the limitations section to more fully acknowledge key methodological and data constraints. In particular, we now discuss (i) the inability of the joint copula framework to directly incorporate survey weights and the implications for national inference, alongside sensitivity analyses; (ii) restriction of joint modeling to children aged 6–59 months due to haemoglobin measurement protocols; (iii) potential malaria misclassification arising from microscopy/RDT limitations and point-prevalence measurement; and (iv) limits to generalizability beyond Uganda given geographic, seasonal, and epidemiological heterogeneity. These additions provide a more transparent assessment of the study’s scope and applicability.

To assess the robustness of our findings, we conducted multiple sensitivity analyses. These included multilevel probit models with PSU-level random effects, and bootstrap-based confidence intervals that preserved clustering and stratification. Across these alternative specifications, the direction and relative magnitude of key associations—particularly the residual dependence between anaemia and malaria and the attenuation of maternal anaemia effects under joint modeling—remained substantively unchanged, supporting the stability of the main conclusions despite methodological constraints.

**Reviewer Comment**

If necessary, the authors should conduct sensitivity analyses to assess the robustness of the joint model results, particularly given the exclusion of survey weights, diagnostic limitations, and other acknowledged constraints.

**Response**

We thank the reviewer for this important suggestion. Sensitivity analyses were conducted to assess the robustness of our findings. Specifically, we (i) estimated alternative outcome-specific generalized linear mixed models (GLMMs) with random intercepts at the primary sampling unit (PSU) and household levels to partially account for clustering, (ii) compared joint-model marginal effects with those from survey-weighted univariate probit models, and (iii) implemented PSU-level, strata-preserving bootstrap procedures to evaluate the stability of the copula dependence parameter and marginal effects. Across these checks, the direction, relative magnitude, and statistical significance of key associations—including child age, region, maternal anaemia status, and the positive residual dependence between malaria and anaemia—were consistent with the main joint Gaussian copula results. We have clarified the placement and interpretation of these sensitivity analyses in the Discussion (Limitations and Robustness subsection) and note that they support the validity of the substantive conclusions despite acknowledged methodological constraints.

**Reviewer Comment**

Inappropriate placement: The simulation is assigned to the Methodology or appendices, not the main Results.

**Response**

We thank the reviewer for this helpful suggestion. We agree that the simulation-based analyses are methodological in nature and are intended to assess the robustness and validity of the joint modeling framework rather than to present primary empirical findings. Accordingly, we have removed the simulation description from the main Results section and relocated it to the Methods, under a dedicated subsection on model evaluation and validation. Full technical details of the simulation design and outcomes have been moved to the Supplementary Materials (S4 File), with only a brief reference retained in the main manuscript. This revision improves clarity, maintains focus on empirical results, and aligns the manuscript structure with PLOS ONE guidelines.

**Reviewer Comment**

The Discussion contains unsupported assumptions (e.g., “common factors such as malnutrition”) that are not directly linked to the study’s results or cited evidence. Several paragraphs are overly general and repetitive, reiterating concepts such as false conditional independence, univariate bias, and copula benefits without adding new insight. Some examples are vague (e.g., maternal anaemia and wealth effects) without reference to specific marginal effects or model differences. The concluding paragraph lacks a clear closing message. In addition, the Strengths and Limitations section is excessively long, repeats methodological advantages already discussed, and presents broad recommendations (e.g., longitudinal data, alternative copulas) without explicit linkage to the study’s findings.

**Response**

We thank the reviewer for this detailed and constructive critique. We have substantially revised and streamlined the Discussion and Strengths and Limitations sections to improve focus, clarity, and evidentiary support. Specifically, we have:

i. Removed or qualified unsupported causal explanations (e.g., malnutrition, access to care) and now explicitly frame these factors as *plausible but unmeasured contributors* to residual dependence, clearly distinguishing interpretation from inference and citing relevant external literature where appropriate.
ii. Reduced repetition across the Discussion, consolidating overlapping paragraphs on conditional independence, univariate bias, and copula advantages into a single, focused synthesis paragraph that directly references our empirical findings (e.g., attenuation and sign changes in maternal anaemia and education effects).
iii. Anchored interpretation to study results, explicitly referring to observed changes in Average Marginal Effects and statistical significance when contrasting separate and joint models, rather than using abstract examples.
iv. Revised the concluding paragraph to provide a clear, evidence-based take-home message that links the estimated residual dependence (ρ = 0.19) and marginal effect stability to concrete implications for epidemiological modeling and integrated child health policy.
v. Condensed the Strengths and Limitations section by more than half, removing repetition of previously stated methodological benefits and restructuring it to focus only on limitations directly arising from this study (e.g., lack of survey weights in joint modeling, age restriction to 6–59 months, diagnostic misclassification, and generalizability beyond Uganda). Recommendations for future research are now explicitly tied to these limitations.

**Reviewer Comment**

In general, the methodology is too long, boring the reader with repetition and unnecessary details. Excessive length, repetition, and detailed information discourage reading and bore the reader. It is recommended to be concise, mentioning only the essential points and those worthy of mention, integrating ideas, and refining the methodology.

**Response**

We thank the reviewer for this constructive comment. We have substantially revised and refined the Methods section to improve clarity, conciseness, and readability.

Specifically, we streamlined the main Methods to focus exclusively on the essential elements required to understand the study design, the proposed joint modeling framework, and its interpretation. Repetitive textbook-style descriptions and extensive technical details were removed from the main text and relocated to the Supplementary Methods. This includes background descriptions of alternative joint modeling approaches, detailed derivations of the maximum simulated likelihood, extended discussions of estimation algorithms, and full technical specifications of robustness analyses.

The revised Methods now emphasize the study-specific modeling choices, the rationale for the proposed Gaussian copula approach, and the key inferential outputs, while maintaining methodological transparency through clearly labeled Supplementary Files (S5–S8). As a result, the length of the main Methods section has been substantially reduced, redundancies eliminated, and the overall flow improved.

Relatedly, results that primarily served a diagnostic or supportive role rather than conveying primary findings were relocated to the Supplementary Results. Preliminary evidence of dependence between anaemia and malaria based on log-linear models is now presented in Supplementary Results S5, while extended comparisons between separate and joint marginal models are reported in Supplementary Results S6. Simulation-based sensitivity analyses and additional model validation exercises have been moved in full to Supplementary Results S7.

As a result, the revised Results section is substantially shorter, avoids repetition of tables and methodological justification, and focuses on reporting key findings from the joint model. The Supplementary Materials provide full technical transparency for interested readers without burdening the main narrative. We believe these changes directly address the reviewer’s concern and have significantly improved the readability and coherence of the manuscript.

**Reviewer Comment**

The striking reversal of the association between maternal anaemia and child malaria risk in the joint model requires deeper discussion, including potential biological and social mechanisms, to justify the interpretation that the joint model corrects bias in the separate model.

**Response**

We appreciate this important observation and have expanded the Discussion to provide a deeper and more nuanced interpretation of this finding. We now explicitly discuss how the reversal likely reflects correction of bias arising from unmeasured shared risk factors—such as nutrition, healthcare access, and socioeconomic vulnerability—that simultaneously affect maternal health, child anaemia, and malaria risk. By accounting for residual dependence between outcomes, the joint model yields estimates that are more biologically and socially plausible, underscoring the importance of modeling comorbidity when maternal and child health determinants are intertwined. This expanded discussion is now included in the revised Discussion section.

**Reviewer 3**

**Reviewer comment**
Line 21: Please use “aims” instead of “seeks” in the sentence “This study seeks to identify…”, as it is more standardized in scientific research and more appropriate in an epidemiological context.

**Response**
Thank you for this helpful suggestion. We agree that “aims” is more standard in epidemiological writing. We have revised the sentence accordingly. The sentence now reads: “This study aims to identify the dependence structure between anaemia and malaria among children under five using a joint modeling approach.”

**Reviewer Comment**
Introduction (Lines 43–45): The paragraph provides a description of the current situation in the research area, but it does not provide any statistical figures or research evidence to support this claim. It would be better to rephrase it and add figures that support this claim, whether from the World Health Organization, the Ugandan Ministry of Health, or even from any reliable and credible published research*.*

**Response:**
Thank you for this helpful suggestion. We have revised the Introduction (Lines 43–45) to include survey-based quantitative evidence supporting the stated burden of malaria and anaemia in Uganda. Specifically, we now report nationally cited figures indicating that malaria remains common among under-five children (approximately ~21% malaria positivity in the 2018–2019 Uganda MIS) and that childhood anaemia remains highly prevalent (53% among children aged 6–59 months, with severity categories reported in the 2016 Uganda DHS). These additions strengthen the epidemiological motivation for the study and provide a clearer empirical basis for the research problem.

**Reviewer Comment**

Lines 45–48: The paragraph explains that anemia affects about half of children under five in the region, and the authors link this to malnutrition, chronic infections, and parasitic diseases. However, there are other causes such as bacterial and viral infections, so “infectious diseases, including malaria” would have been better for greater comprehensiveness.

**Response**

We thank the reviewer for this important clarification. We have revised Lines 45–48 to adopt more comprehensive language by referring to infectious diseases broadly, including malaria as well as bacterial and viral infections, alongside malnutrition, parasitic infestations, and chronic inflammation. This revision better reflects the multifactorial aetiology of childhood anaemia in high-burden settings and improves the epidemiological accuracy and completeness of the Introduction.

**Reviewer Comment**

Lines 74–76: This paragraph lacks a reference. Please add a reliable reference.

**Response**

Thank you for this observation. We have revised the manuscript to address this concern by adding appropriate and up-to-date citations to support the statements made in Lines 74–76. Specifically, we have incorporated references from peer-reviewed literature and international public health sources that document the burden and determinants of childhood anaemia and malaria in sub-Saharan Africa and Uganda. These additions ensure that all claims in the paragraph are now fully supported by credible empirical evidence.

**Reviewer Comment**

Lines 80–83: The paragraph is more suited to the methodology section and should be moved there. It was also noted that the paragraphs at the end of the Introduction lack references and are generally inconsistent. This should be reviewed and addressed as needed.

**Response**

We thank the reviewer for this constructive comment. In response, we have carefully revised the Introduction section as follows.

First, the paragraph in Lines 80–83, which described aspects of the analytical approach, has been removed from the Introduction and relocated to the *Statistical Analysis* subsection of the Methods, where it more appropriately belongs. This restructuring improves the conceptual flow of the manuscript by ensuring that the Introduction focuses on background, motivation, and study aims, while methodological details are confined to the Methods section.

Second, we thoroughly reviewed the concluding paragraphs of the Introduction to address the noted inconsistencies and missing references. We have revised the text for clarity and coherence and added relevant, up-to-date citations to support all substantive claims. These revisions ensure that the Introduction is now internally consistent, well-referenced, and aligned with standard epidemiological writing conventions.

**Reviewer Comment**

The paragraphs from line 77 to the end of the Introduction aim to highlight the importance and purpose of the study. However, this section is overly long and repetitive. These paragraphs should be rewritten and substantially shortened.

**Response**

We thank the reviewer for this helpful suggestion. We agree that the concluding portion of the Introduction was overly detailed. In response, we have substantially revised and condensed the paragraphs from line 77 to the end of the Introduction. The revised text now provides a concise and focused statement of the study’s motivation, aims, and contribution, while avoiding redundancy and excessive methodological detail. This restructuring improves readability and ensures that the Introduction remains succinct and aligned with standard epidemiological writing conventions.

**Reviewer Comment (Lines 92–93):**
The UMIS 2018–2019 statistics are the most recent national survey data on malaria and anemia in Ugandan children. However, the fact that several years have elapsed since the survey may restrict the data’s applicability to the present epidemiological situation, particularly in light of potential changes in control measures and disease burden.

**Response:**
We thank the reviewer for this important observation. We acknowledge that the UMIS 2018–2019 data reflect the epidemiological situation at the time of the survey and that subsequent changes in malaria control strategies and broader health interventions may have influenced current malaria and anaemia patterns in Uganda. We have now explicitly acknowledged this temporal limitation in the **Strengths and Limitations** section of the Discussion, noting that while the data remain the most recent nationally representative source with concurrent malaria and haemoglobin measurements, the findings should be interpreted as reflective of the 2018–2019 period rather than current prevalence levels. Importantly, our primary objective is methodological—demonstrating the value of joint modeling for correlated childhood outcomes—rather than providing contemporaneous prevalence estimates. The modeling framework and insights into residual dependence remain relevant and applicable to more recent and future datasets as they become available.

**Reviewer Comment (Lines 121–126)**

The description of data linking in paragraphs 121–126 is technically clear but overly brief and lacks supporting quantitative evidence, thus diminishing its scientific credibility. While mentioning screening, exclusion, and representativeness verification, it fails to specify the percentage of records excluded, the statistical tests used to compare demographic variables, or the main variables compared, rendering the claim of “no bias” quantitatively unsupported. Adding a sentence containing figures or a before/after comparison table would enhance transparency and methodological reliability.

**Response**

We thank the reviewer for this important methodological clarification. We agree that the original description of the data linkage process lacked quantitative detail. In response, we have expanded the Data Linkage section to include the proportion of records excluded during merging, the key demographic variables assessed for representativeness, and the statistical tests used to compare pre- and post-linkage samples. These additions strengthen transparency and provide quantitative support for the claim that no systematic bias was introduced during data linkage.

**Reviewer Comment**

Lines 161–162: It is best to maintain complete consistency throughout the manuscript by using either “children under five years of age” or “children aged 0–59 months,” rather than alternating between the two.

**Response**

We thank the reviewer for highlighting this important issue of consistency. We agree that inconsistent age definitions can reduce clarity and coherence. We have revised the manuscript to use “under five years” consistently throughout the Methods, Results, Tables, and Discussion, while retaining “children aged 0–59 months” only where used descriptively or in titles as a synonymous expression. This harmonization ensures methodological precision and alignment with DHS/MIS reporting standards.

**Reviewer Comment (Lines 192–362)**

The methodology section is excessively long (>150 lines), repetitive in its presentation of basic concepts (e.g., Y notation, probit models), unfocused due to lengthy overviews of GEE/GLMM without direct application, contains incomplete equations (9–10) without explanation of S in MSL, and includes scattered diagnostic checks (VIFs/GLMM/bootstrap) without clear results. The reviewer recommends dividing the section into justification, equations, and evaluation, with detailed material moved to appendices or comparison tables.

**Response**
We appreciate this detailed and constructive critique and have substantially revised the Methods section to address all concerns raised. Specifically:

1. Length and repetition: We have removed repetitive textbook-style explanations of basic concepts (e.g., binary outcome notation, probit fundamentals) and retained only material essential for understanding the study-specific modeling choices.
2. Focus and structure: The Methods have been reorganized into three clearly defined subsections: (i) *Model justification and rationale*, (ii) *Core model specification and estimation*, and (iii) *Model evaluation and robustness checks*. This restructuring improves logical flow and readability.
3. Unfocused overviews: Extended discussions of GEE and GLMM frameworks without direct application have been removed from the main text and relocated to the Supplementary Methods, where they are briefly referenced as alternative or sensitivity approaches.
4. Incomplete equations: All truncated equations have been corrected, and the role of the simulation parameter *S* in the maximum simulated likelihood (MSL) procedure is now explicitly defined and explained.
5. Diagnostic and robustness checks: Checks such as VIF assessment, GLMM sensitivity analyses, and bootstrap procedures are now summarized succinctly in the main Methods, with detailed results and diagnostics moved to the Supplementary Materials and clearly referenced.

**Reviewer Comment (Lines 356–370)**

Lines 356–370 contain the same text as the table above; therefore, the table is unnecessary as it is redundant and adds nothing new.

**Response**

We agree with the reviewer that the text duplicated the information presented in the table. In the revised manuscript, we have removed the repetitive narrative description and retained only the table to present prevalence estimates concisely. The Results section now briefly references the table without restating numerical values, improving readability and avoiding redundancy.

**Reviewer Comment (Lines 371–390)**

This section is overly detailed and unfocused, repeating the log-linear equation twice (17, 18), including an unnecessary expanded form, and presenting a detailed table without sufficient statistical context (e.g., fit statistics, interpretation of OR = 2.64, or sample size). It would have been better summarized in a short paragraph.

**Response**

We appreciate this suggestion and have substantially revised this section. The repeated equations and expanded algebraic form were removed, and the detailed log-linear table was relocated to the Supplementary Results. In the main text, the section has been condensed to a single summary paragraph, now stating that the homogeneous log-linear model provided the best fit and that the anaemia–malaria interaction was statistically significant (OR = 2.64, p < 0.001), thereby confirming dependence prior to the copula analysis. Additional model diagnostics and parameter details are now clearly presented in the Supplementary Materials.

**Reviewer Comment (Lines 393–411)**

These paragraphs are repetitive and out of context, reiterating methodological justification (“Why copula?”) already discussed earlier, while promising results that are not presented here.

**Response**

We agree with this assessment. These paragraphs have been removed from the Results section and either deleted or relocated to the Methods as preliminary diagnostic motivation, depending on content. The Results section now strictly reports empirical findings, while methodological justification is confined to the Methods. This restructuring eliminates redundancy and restores a clear Results–Methods separation.

**Reviewer Comment (Lines 419–455)**

This section is overly detailed, lacks conciseness, repeats interpretations across Tables 5 and 6 without quantitative figures, and reads more like a Discussion than Results.

**Response**

We thank the reviewer for this constructive guidance. This section has been substantially shortened and refocused. Detailed interpretive language (e.g., “more realistic,” “bias correction”) has been removed from the Results and deferred to the Discussion. The revised Results now provide a brief comparative summary highlighting key covariates whose effects differed between separate and joint models, with direct references to Tables 5 and 6. Extended explanations and implications are now addressed exclusively in the Discussion.

**Reviewer Comment (Lines 470–514)**

Simulation results are inappropriately placed in the Results section; claims of “strong support” lack transparency; and key validation statistics are omitted.

**Response**

We fully agree. The simulation study has been moved in its entirety to the Supplementary Results, where full details on bias, confidence interval coverage, and robustness are provided. In the main Results section, we now include a concise validation summary, reporting key statistics from the empirical model (copula correlation estimate with 95% CI, AUC values for anaemia and malaria, and model fit criteria). This revision improves transparency while keeping the main Results focused.

**Global Comment on Results Organization**

The Results are scattered, lengthy, and blur the boundary between Results, Methods, and Discussion.

**Response**

We acknowledge this concern and have undertaken a complete reorganization of the Results section. The revised structure now follows a clear sequence: (i) prevalence and co-occurrence, (ii) residual dependence estimates, (iii) comparison of marginal effects, and (iv) concise model validation. Methodological justification and extended analyses have been relocated to the Methods or Supplementary Materials, substantially improving coherence and conciseness.

**Reviewer Comment (Lines 520–527)**

The contribution is vague and unsupported because references to previous studies are not explicitly mentioned.

**Response**

We agree and have revised this paragraph to explicitly reference prior joint modeling studies in similar contexts. The revised text now clearly situates our contribution relative to existing literature, strengthening the comparative claim.

**Reviewer Comment (Lines 527–530)**

Unsupported assumptions are made regarding common factors such as malnutrition without linking to study findings.

**Response**

This paragraph has been revised to clearly frame such factors as plausible explanations, explicitly noting that they were not directly measured in the present analysis. Overly speculative language has been removed, and interpretations are now more tightly linked to the observed residual dependence.

**Reviewer Comment (Lines 543–549)**

The concluding paragraph is general and not supported by specific evidence.

**Response**

We have revised the conclusion to explicitly reference key empirical findings, including the estimated copula correlation and observed changes in marginal effects under joint modeling, thereby grounding the summary in concrete results.

**Reviewer Comment (Lines 566–591)**

This section contains excessive repetition, vague examples, and ends abruptly.

**Response**

We have condensed this section substantially, eliminating repeated explanations of conditional independence and joint modeling advantages. Examples are now explicitly tied to results reported in Tables 5 and 6, and the section now concludes with a clear synthesis linking statistical findings to substantive implications.

**Reviewer Comment (Lines 593–636)**

The Strengths and Limitations section is excessively long and repetitive, with generic future recommendations.

**Response**

We agree and have restructured this section into a concise strength–limitations format, reducing length by more than half. Repetitive methodological praise has been removed, and future directions are now directly motivated by limitations identified in this study, improving focus and relevance.

Sincerely,

**Grace Kakaire**

on behalf of all authors
