# Supplementary material for: Co-occurrence of anaemia and malaria among children under five in Uganda: A joint modeling analysis
Source: PLOS Glob Public Health. 2026 Oct 1;6(10):e0007387. doi: 10.1371/journal.pgph.0007387 (PMC13630190; doi:10.1371/journal.pgph.0007387)
Supplement: Supplementary file 16 [file pgph.0007387.s017.docx]

**Rebuttal to Reviewer and Editor Comments**

**Manuscript title:** Co-occurrence of Anaemia, Fever, and Malaria Among Children Under Five in Uganda: A Joint Modeling Analysis

**Manuscript ID:** PGPH-D-25-01028

Dear Editor,

We thank you and the reviewer for the thoughtful feedback. Below, we address the comments point by point. All changes are reflected in the revised manuscript

**Reviewer Comment (Lines 52–57):**
The paragraph requires clarification in several areas. First, the statement that malaria is one of the leading causes of child mortality should be supported with recent epidemiological data from reliable sources (e.g., WHO). Second, the distinction between infectious diseases and parasitic infestations is conceptually redundant, as parasitic infections are part of infectious diseases; the wording should be revised accordingly. Finally, the sentence structure describing the multifactorial aetiology of anaemia needs grammatical correction and clearer formulation.

**Response:**
We thank the reviewer for this insightful comment. We have revised the paragraph to improve clarity, conceptual accuracy, and grammatical structure.

First, we have strengthened the statement regarding malaria as a leading cause of child mortality by incorporating recent epidemiological evidence from the World Health Organization (WHO), specifically noting the disproportionate burden of malaria-related deaths among children under five years of age.

Second, we have addressed the conceptual redundancy by removing the separate reference to “parasitic infestations” and instead referring more broadly to “infectious diseases (including malaria and other infections),” which more accurately reflects the classification of parasitic infections within infectious diseases.

Finally, we have revised the sentence describing the multifactorial aetiology of anaemia to improve grammatical clarity and readability. The revised text now presents the contributing factors—malnutrition, infectious diseases, and chronic inflammation—in a more concise and logically structured manner.

**Reviewer Comment:**
The sentence “What is known from existing literature is that…” is stylistically weak and unnecessarily indirect for scientific writing. Academic writing typically favors more direct statements supported by citations.

**Response:**
We thank the reviewer for this helpful observation. We agree that the phrasing was unnecessarily indirect. The sentence has been revised to a more direct and concise form, removing the introductory clause and presenting the evidence more clearly. The revised text now begins with a direct statement of the established relationship between anaemia and malaria, supported by appropriate citations. This change improves the clarity and academic tone of the manuscript.

**Reviewer Comment:**
The introduction should be revised to incorporate information on malaria and anemia, their association, and the identification of risk factors in Uganda. The study's objectives and justifications should also be briefly stated.

**Response:**
We thank the reviewer for this constructive suggestion. In response, we have revised the Introduction to more clearly integrate evidence on malaria and anaemia, their association, and key risk factors within the Ugandan context. Specifically, we have strengthened the discussion of shared biological and socioeconomic determinants, including household wealth, maternal education, sanitation, and access to health services.

In addition, we have clarified the linkage between malaria and anaemia by explicitly describing the biological and epidemiological pathways through which malaria contributes to anaemia.

Finally, we have revised the concluding section of the Introduction to explicitly state the study objectives and justification. The revised text now clearly outlines the aim of applying a joint modeling framework to assess the dependence between anaemia and malaria among children under five, and highlights the relevance of this approach for informing integrated child health interventions in Uganda. These revisions improve the clarity, coherence, and overall focus of the Introduction.

**Reviewer Comment:**
The paragraphs are rich in information but lack a smooth flow from study design to statistical analysis. It would be better to divide the section into clear headings: Study area; Data Source and Sample Design; Sample Selection and Preparation; Defining Variables and Measurements; Statistical Model and Analysis; Ethical Considerations. The scientific approach is good, but it needs a greater focus on rationale rather than minor technical details.

**Response:**
We thank the reviewer for this valuable suggestion. We agree that the Materials and Methods section would benefit from improved structure and clearer progression from study design to statistical analysis.

In response, we have reorganized the section into clearly defined subsections: *Study Area; Data Source and Sample Design; Sample Selection and Preparation; Defining Variables and Measurements; Statistical Model and Analysis; and Ethical Considerations*. This restructuring improves readability and ensures a logical flow of information.

In addition, we have revised the text to place greater emphasis on the rationale underlying key methodological choices—particularly the selection of the joint modeling framework—while reducing excessive technical detail in the main text. Detailed derivations and computational procedures have been moved to the Supplementary Materials.

These changes enhance the clarity, coherence, and accessibility of the Methods section without compromising scientific rigor.

**Reviewer Comment:**
The manuscript reports that “21.720% had anaemia only”, but the total number of participants corresponding to this percentage is not provided. In epidemiological reporting, percentages should always be accompanied by the absolute number of cases (n) and the total sample size (N) to allow readers to properly interpret the magnitude of the finding.

**Response:**
We thank the reviewer for this important observation. We agree that reporting both percentages and corresponding absolute counts improves clarity and interpretability.

In response, we have revised the Results section to report all prevalence estimates with both the number of cases (n) and the total sample size (N). Specifically, the statement has been updated to include the absolute counts alongside percentages for each category of malaria and anaemia combinations. These revisions ensure consistency with epidemiological reporting standards and improve transparency of the findings.

**Reviewer Comment:**
Table 2 (Prevalence of Malaria and Anaemia Combinations) appears to present information that has already been described in detail in the preceding text. This results in unnecessary redundancy and does not add additional analytical value. In scientific reporting, tables should provide new insights, clearer comparisons, or more detailed data that cannot be efficiently presented in the narrative text.

**Response:**
We thank the reviewer for this important observation. We agree that redundancy between the narrative text and Table 2 should be minimized.

In response, we have revised the Results section to streamline the text while retaining Table 2 as a complementary element. Specifically, the narrative now presents the key prevalence findings concisely and includes a direct reference to Table 2, rather than repeating the full set of detailed values. The table has been retained to provide a structured presentation of the complete distribution of malaria–anaemia combinations, including absolute counts, percentages, and confidence intervals, which would be less efficiently conveyed in the text alone.

**Reviewer Comment:**

Lines 287–282. The paragraph beginning with “As discussed, we fitted two sets of models: separate marginal probit regressions and a joint Gaussian copula regression model” includes methodological explanations that are not appropriate for the Results section. The Results section should focus strictly on presenting the findings of the analysis, without reiterating methodological details or providing interpretation of the modeling strategy. Statements describing the modeling framework, assumptions (e.g., conditional independence), and the rationale for using a joint copula model belong in the Methods section, while interpretation of the findings should appear in the Discussion.

**Response:**

We thank the reviewer for this helpful comment. We agree that the original paragraph included methodological detail that is better placed in the Methods section.

In response, we have revised the Results section by removing the explanatory text describing the separate marginal probit models, the joint Gaussian copula framework, and the rationale for its use. These methodological details have been retained in the Methods section, while the Results section now focuses only on the empirical findings, including the estimated residual correlation and the comparative results from the separate and joint models. Interpretive discussion of the implications of these findings has also been reduced in the Results section and reserved for the Discussion.

Lines 283–287. The paragraph beginning with “To assess whether a joint modeling approach was necessary…” contains methodological reasoning and justification for selecting the joint Gaussian copula regression model. Such explanations are more appropriate for the Methods section, rather than the Results section.

**Response:**

We thank the reviewer for this helpful observation. We agree that the paragraph included methodological reasoning that is more appropriately presented in the Methods section.

In response, we have revised the manuscript by removing this explanatory text from the Results section. The rationale for selecting the joint Gaussian copula regression model, including the assessment of dependence between outcomes, has been incorporated into the Methods section. The Results section has been streamlined to focus solely on the empirical findings, including the estimated correlation and model outputs, without methodological justification.

**Reviewer Comment (Points 5–7):**

Lines 288–297 and 305–310 contain methodological explanations and interpretative commentary that are not appropriate for the Results section. More broadly, the Results section shows weaknesses in clarity and scientific structure, with findings mixed with methodological details and interpretation. Additionally, some tables repeat information already described in the text rather than adding analytical value.

**Response:**

We thank the reviewer for these comprehensive and constructive comments. We agree that the Results section required clearer separation between empirical findings, methodological description, and interpretation.

In response, we have undertaken a substantial revision of the Results section. First, all methodological explanations—including descriptions of model assumptions, justification for the joint modeling framework, and estimation procedures—have been removed from the Results and consolidated within the Methods section.

Second, interpretative and explanatory statements have been minimized in the Results section and reserved for the Discussion. The revised Results now focus strictly on presenting the empirical findings in a clear and neutral manner, including prevalence estimates, model outputs, and measures of association.

Third, the structure of the Results section has been reorganized to improve logical flow, progressing from descriptive statistics to model-based findings and comparative results.

For example, paragraphs previously describing the rationale for the joint modeling approach have been removed from the Results section, and replaced with concise statements reporting the estimated copula correlation and model outputs. Similarly, redundant textual repetition of Table 2 has been reduced by summarizing key findings and referencing the table for detailed values.

**Reviewer Comment:**
Line 362. The phrase “In this paper, this study…” is redundant and stylistically weak. The use of both “paper” and “study” conveys the same meaning and results in unnecessary repetition.

**Response:**
We thank the reviewer for this helpful observation. We agree that the phrasing was redundant and stylistically weak. The sentence has been revised to remove the duplication and improve clarity and conciseness. The revised text now uses a more direct formulation consistent with standard academic writing style.

**Reviewer Comment:**
The discussion repeats too many results that were already presented in the Results section. The authors should summarize only the most important findings.

**Response:**
We thank the reviewer for this important observation. We agree that the Discussion section should focus on synthesizing and interpreting the key findings rather than repeating detailed results.

In response, we have revised the Discussion to reduce redundancy by removing repetitive descriptions of results already presented in the Results section. The revised text now highlights only the most important findings—particularly the co-occurrence of anaemia and malaria, the estimated residual dependence between the two conditions, and key differences between joint and separate models—while emphasizing their interpretation and relevance.

Grace Kakaire

On behalf of all authors
