# Supplementary material for: Co-occurrence of anaemia and malaria among children under five in Uganda: A joint modeling analysis
Source: PLOS Glob Public Health. 2026 Oct 1;6(10):e0007387. doi: 10.1371/journal.pgph.0007387 (PMC13630190; doi:10.1371/journal.pgph.0007387)
Supplement: Supplementary file 17 [file pgph.0007387.s018.docx]

Dear Kakaire,

*Joint Modeling of Childhood Illnesses in Uganda*

This is a well-constructed manuscript that addresses a critical gap in pediatric epidemiology: the over-reliance on univariate models for co-occurring conditions. I find the application of *Gaussian copula-based joint modeling* to be a statistically robust way to handle the "syndemic" nature of malaria and anaemia. I specify the strength of this manuscript as below

1. Core Concept and Significance

The study moves beyond the standard assumption of conditional independence—a frequent flaw in epidemiological literature. The appropriate usage of bivariate probit model in this study has proven the authors correct choice in identifying the residual correlation of 0.19 between malaria and anaemia to be not merely a product of shared observed covariates but also latent biological and environmental factors (e.g., unmeasured nutritional status or immunological).

2. Methodological Rigor

- *The Copula Framework*: The choice of a Gaussian copula is appropriate for binary margins. It allows for the separation of the marginal distributions (the probability of each disease) from the dependence structure.
- *Statistical Implementation*: The use of the GJRM package in R is a modern, high-standard choice for generalized joint regression. I particularly appreciate the authors' transparency regarding the limitations of survey-weighting within GJRM and their subsequent sensitivity analyses using PSU-level random effects to ensure stability.
- *Average Marginal Effects (AMEs)*: Reporting AMEs rather than raw probit coefficients is essential for public health interpretability, as it translates latent variables into actionable probability shifts.

3. Key Findings & Biological Plausibility

The most striking result is the *sign reversal* of maternal anaemia’s effect on child malaria risk when moving from a separate to a joint model. In the separate model, the association appeared counterintuitive; the joint model corrected this, likely by accounting for the endogeneity of the maternal-child health environment. This highlights how independent models can mislead policy by misattributing risk.

4. Critical Limitations to Address

While the manuscript is strong, there are two areas where I would push for clearer nuance:

- *Temporal Mismatch*: Malaria microscopy provides a "snapshot" of current infection, whereas anaemia (haemoglobin levels) reflects a longer physiological history. The authors mention this, but the discussion could more explicitly state that the 0.19 correlation might actually be an *underestimate* due to this temporal lag.
- *Multifactorial Aetiology*: The model correctly identifies that non-malarial causes (iron deficiency, helminths) attenuate the correlation. However, the study could benefit from a brief discussion on how future models might integrate nutritional biomarkers if they were available in the MIS.
- *Figures refinements*: Ensure the flowchart for the Gaussian copula estimation is high-resolution. I struggled to see clearly.

Decision: ACCEPT (with minor editorial polish)

This is a "must-read" for researchers looking to bridge the gap between dynamical systems theory and empirical surveillance data.
