# Supplementary material for: Co-occurrence of anaemia and malaria among children under five in Uganda: A joint modeling analysis
Source: PLOS Glob Public Health. 2026 Oct 1;6(10):e0007387. doi: 10.1371/journal.pgph.0007387 (PMC13630190; doi:10.1371/journal.pgph.0007387)
Supplement: Supplementary file 18 [file pgph.0007387.s019.docx]

**Rebuttal to Reviewer and Editor Comments**

**Manuscript title:** Co-occurrence of Anaemia and Malaria Among Children Under Five in Uganda: A Joint Modeling Analysis

**Manuscript ID:** PGPH-D-25-01028

Dear Editor,

Response to Reviewer

We sincerely thank the reviewer for the careful assessment of our manuscript, “Joint Modeling of Childhood Illnesses in Uganda,” and for the positive evaluation of its methodological contribution, statistical implementation, and public-health relevance. We particularly appreciate the reviewer’s recognition of the value of jointly modeling anaemia and malaria and of our efforts to assess the robustness of the findings despite the limitations associated with incorporating the complex survey design directly within the joint modeling framework.

We have carefully considered the reviewer’s suggestions and revised the manuscript accordingly. Our point-by-point responses are provided below.

Comment 1:

Malaria microscopy provides a snapshot of current infection, whereas anaemia measured using haemoglobin reflects a longer physiological history. The discussion should more explicitly acknowledge that the estimated correlation may be attenuated by this temporal mismatch.

Response:

We thank the reviewer for highlighting this important point. We agree that malaria microscopy and haemoglobin measurements represent different temporal dimensions of child health. Microscopy identifies parasitaemia at the time of the survey, whereas haemoglobin concentration may reflect cumulative or recent influences from malaria as well as nutritional deficiencies, other infections, inflammation, and other causes of anaemia.

We have therefore expanded the Discussion to make this temporal mismatch more explicit. We have, however, avoided concluding definitively that the estimated residual correlation of ρ = 0.191 is an underestimate, because the cross-sectional data do not allow us to determine the direction or magnitude of any resulting attenuation. Instead, we now state that temporal discordance between the two measurements may attenuate the observed contemporaneous dependence and should be considered when interpreting the estimated residual association.

Comment 2: Multifactorial aetiology and nutritional biomarkers

The study could benefit from a brief discussion of how future models might incorporate nutritional biomarkers, given that anaemia has important non-malarial causes such as iron deficiency and helminth infection.

Response:

We agree with the reviewer. Anaemia is a multifactorial condition, and the available MIS variables cannot capture all biological and nutritional pathways underlying childhood anaemia. We have expanded the limitations and future-research discussion to acknowledge this issue and to indicate how richer biomarker data could strengthen future joint modeling studies.

Comment 3: Figure/flowchart resolution

Ensure that the flowchart for the Gaussian copula estimation is high-resolution because the current version is difficult to read.

Response:

Thank you for pointing this out. We have revised the Gaussian copula methodological flowchart to improve its readability and presentation. The figure has been regenerated at higher resolution, with enlarged text, improved spacing, and clearer connections between the marginal probit models, Gaussian copula dependence structure, estimation procedure, and model evaluation components. The revised high-resolution figure has been incorporated into the manuscript.

Additional editorial refinement

We also reviewed the manuscript to ensure that the interpretation of the copula dependence parameter remains appropriately cautious. The estimated ρ = 0.191 is now consistently described as a small positive residual/latent association between anaemia and malaria conditional on the included covariates, rather than as direct evidence of a specific unmeasured biological mechanism. This distinction is important because the cross-sectional survey does not directly identify the unobserved factors generating the residual dependence.

We again thank the reviewer for the constructive comments and positive assessment. We believe that these revisions have improved the clarity of the manuscript, particularly regarding the temporal interpretation of the malaria and anaemia measurements, the multifactorial nature of childhood anaemia, and the presentation of the joint modeling framework.

Grace Kakaire

On behalf of all authors
