# Supplementary material for: Co-occurrence of anaemia and malaria among children under five in Uganda: A joint modeling analysis
Source: PLOS Glob Public Health. 2026 Oct 1;6(10):e0007387. doi: 10.1371/journal.pgph.0007387 (PMC13630190; doi:10.1371/journal.pgph.0007387)
Supplement: Peer review history

# Peer review: Decision Letter 0

**DOI:** 10.1371/journal.pgph.0007387.r001

**Authors:** Jason Morgan

29 Aug 2025

PGPH-D-25-01028

Co-occurrence of Anaemia, Fever, and Malaria Among Children Under Five in Uganda: A Joint Modeling Analysis

PLOS Global Public Health

Dear Dr. Kakaire,

Thank you for submitting your manuscript to PLOS Global Public Health. After careful consideration, we feel that it has merit but does not fully meet PLOS Global Public Health’s publication criteria as it currently stands. Therefore, we invite you to submit a revised version of the manuscript that addresses the points raised during the review process.

Please note that we have only been able to secure a single reviewer to assess your manuscript. We are issuing a decision on your manuscript at this point to prevent further delays in the evaluation of your manuscript. Please be aware that the editor who handles your revised manuscript might find it necessary to invite additional reviewers to assess this work once the revised manuscript is submitted. However, we will aim to proceed on the basis of this single review if possible.

Please submit your revised manuscript by Oct 10 2025 11:59PM. If you will need more time than this to complete your revisions, please reply to this message or contact the journal office at globalpubhealth@plos.org. When you're ready to submit your revision, log on to https://www.editorialmanager.com/pgph/ and select the 'Submissions Needing Revision' folder to locate your manuscript file.

Please include the following items when submitting your revised manuscript:

- A rebuttal letter that responds to each point raised by the editor and reviewer(s). You should upload this letter as a separate file labeled 'Response to Reviewers'.
- A marked-up copy of your manuscript that highlights changes made to the original version. You should upload this as a separate file labeled 'Revised Manuscript with Track Changes'.
- An unmarked version of your revised paper without tracked changes. You should upload this as a separate file labeled 'Manuscript'.

Guidelines for resubmitting your figure files are available below the reviewer comments at the end of this letter.

We look forward to receiving your revised manuscript.

Kind regards,

Dr Jason Morgan

Staff Editor

PLOS Global Public Health

Journal Requirements:

1. Please note that PLOS Global Public Health has specific guidelines on code sharing for submissions in which author-generated code underpins the findings in the manuscript. In these cases, all author-generated code must be made available without restrictions upon publication of the work. Please review our guidelines at https://journals.plos.org/globalpublichealth/s/materials-and-software-sharing#loc-sharing-code and ensure that your code is shared in a way that follows best practice and facilitates reproducibility and reuse.

If the reviewer comments include a recommendation to cite specific previously published works, please review and evaluate these publications to determine whether they are relevant and should be cited. There is no requirement to cite these works unless the editor has indicated otherwise.

Additional Editor Comments (if provided):

[Note: HTML markup is below. Please do not edit.]

Reviewers' comments:

Reviewer’s Responses to Questions

**Comments to the Author**

1. Does this manuscript meet PLOS Global Public Health’s publication criteria?

Reviewer #1: Yes

**********

2. Has the statistical analysis been performed appropriately and rigorously?

Reviewer #1: I don't know

**********

3. Have the authors made all data underlying the findings in their manuscript fully available (please refer to the Data Availability Statement at the start of the manuscript PDF file)?

The PLOS Data policy

Reviewer #1: No

**********

4. Is the manuscript presented in an intelligible fashion and written in standard English?

Reviewer #1: Yes

**********

Reviewer #1: The paper deals with an important public health issue of associated morbidities in under-five children in Sub-Saharan Africa. However, the paper involves higher level of statistics, which might be more appropriate for the readers of a journal of statistics. Some comments to improve the quality of the manuscript are as follows.

1. In the methodology, there is no mention of any stratification adopted.

2. There is no mention about the sampling weights.

3. Details about how much missing data, non-response rate, and how it was handled could be added.

4. It was not clear what methodology was adopted to select this sample out of the total dataset.

5. How was fever-related data collected? Self-reported fever and/or assessment using any valid instrument (thermometer)?

6. It is preferred to mention the validation of the model in terms of either Loglikelihood ratio, Pseudo R2, AIC/BIC, or any other estimate.

7. Elaboration on GLMM will be preferred, which variables were considered to have a fixed effect, and the random effect can be mentioned.

8. Confidence Interval for Prevalence Estimates can be provided.

9. Rationale should be a part of the introduction, not the results.

10. Divergent results between the separate model and the combined model could have been discussed with detailed literature support.

11. Correlation can be expressed as low/moderate or high (based on any of the available classification systems), instead of reporting as statistically significant

**********

what does this mean?). If published, this will include your full peer review and any attached files.

**Do you want your identity to be public for this peer review?** If you choose “no”, your identity will remain anonymous but your review may still be made public.

For information about this choice, including consent withdrawal, please see our Privacy Policy

Reviewer #1: No

**********

[NOTE: If reviewer comments were submitted as an attachment file, they will be attached to this email and accessible via the submission site. Please log into your account, locate the manuscript record, and check for the action link "View Attachments". If this link does not appear, there are no attachment files.]

While revising your submission, please upload your figure files to the Preflight Analysis and Conversion Engine (PACE) digital diagnostic tool, https://pacev2.apexcovantage.com/. PACE helps ensure that figures meet PLOS requirements. To use PACE, you must first register as a user. Registration is free. Then, login and navigate to the UPLOAD tab, where you will find detailed instructions on how to use the tool. If you encounter any issues or have any questions when using PACE, please email PLOS at figures@plos.org. Please note that Supporting Information files do not need this step.

# Author comment: Author response to Decision Letter 1

**DOI:** 10.1371/journal.pgph.0007387.r002

24 Oct 2025

Attachment Submitted filename: Rebuttal_Letter.docx

# Peer review: Decision Letter 1

**DOI:** 10.1371/journal.pgph.0007387.r003

**Authors:** Helen Howard

4 Dec 2025

PGPH-D-25-01028R1

Co-occurrence of Anaemia and Malaria Among Children Under Five in Uganda: A Joint Modeling Analysis

PLOS Global Public Health

Dear Dr. Kakaire,

Thank you for submitting your manuscript to PLOS Global Public Health. After careful consideration, we feel that it has merit but does not fully meet PLOS Global Public Health’s publication criteria as it currently stands. Therefore, we invite you to submit a revised version of the manuscript that addresses the points raised during the review process.

Please submit your revised manuscript by Jan 17 2026 11:59PM. If you will need more time than this to complete your revisions, please reply to this message or contact the journal office at globalpubhealth@plos.org. When you're ready to submit your revision, log on to https://www.editorialmanager.com/pgph/ and select the 'Submissions Needing Revision' folder to locate your manuscript file.

Please include the following items when submitting your revised manuscript:

- A rebuttal letter that responds to each point raised by the editor and reviewer(s). You should upload this letter as a separate file labeled 'Response to Reviewers'.
- A marked-up copy of your manuscript that highlights changes made to the original version. You should upload this as a separate file labeled 'Revised Manuscript with Track Changes'.
- An unmarked version of your revised paper without tracked changes. You should upload this as a separate file labeled 'Manuscript'.

Guidelines for resubmitting your figure files are available below the reviewer comments at the end of this letter.

We look forward to receiving your revised manuscript.

Kind regards,

Helen Howard

Staff Editor

PLOS Global Public Health

Journal Requirements:

1. If the reviewer comments include a recommendation to cite specific previously published works, please review and evaluate these publications to determine whether they are relevant and should be cited. There is no requirement to cite these works unless the editor has indicated otherwise.

Additional Editor Comments (if provided):

[Note: HTML markup is below. Please do not edit.]

Reviewers' comments:

Reviewer’s Responses to Questions

**Comments to the Author**

Reviewer #2: (No Response)

**********

publication criteria?

Reviewer #2: Partly

**********

3. Has the statistical analysis been performed appropriately and rigorously?

Reviewer #2: Yes

**********

4. Have the authors made all data underlying the findings in their manuscript fully available (please refer to the Data Availability Statement at the start of the manuscript PDF file)?

The PLOS Data policy

Reviewer #2: Yes

**********

5. Is the manuscript presented in an intelligible fashion and written in standard English?

Reviewer #2: Yes

**********

Reviewer #2: General Comments

Thank you for inviting me to review a manuscript entitled “Co-occurrence of Anaemia and Malaria Among Children Under Five in Uganda: A Joint Modelling Analysis”. This is an interesting study, and the authors have collected a good dataset using the critical advantage methodology. The paper is generally well written and structured. However, from my point of view, the manuscript has some shortcoming with respect to some dataset analyses and text, and I feel this good dataset has not been used to its full extent. Underneath, the reviewer has provided several remarks on the text, as it is often vague and long-winded. In several examples, I also recommended citing more relevant and recent literature. Moreover, the reviewer has made additional comments for more in-depth analyses of the dataset. I have assessed the manuscript, and I can hardly suggest it for publication for the following reasons:

Key critical points are:

a) Please check the grammar carefully in the overall manuscript; somewhere it’s written in the past and somewhere in the present and some here in starting in the paragraph in small letter and also some here written capital letter in the middle. Check the spelling mistakes; there are major spelling errors in the overall document. If possible, please I suggest that this manuscript be reviewed by an English-language expert.

Specific Comments

1. The Background section on the body of manuscript: this section needs revision so that it will cover the nature of the core problem, the known aspect of problem (i.e., what is known about the core problem), the unknown aspect of the problem (What is unknown about the core problem), and the expectations from the current study (what and how the current study will add to the existing knowledge).

2. What type of statistical software do you use (i.e., R, STATA, SAS…) in your analysis and please upload as supplementary material the codes/commands of the statistical software?

3. In page 7 and line 130-131“Children aged 0–59 months were tested for both anaemia and malaria with the parents' or guardians’ consent”? is Children aged 0–59 months??? Is it possible?

4. Please use the latest reference rather than old reference! Like reference 2, 3, 21 etc.…

5. Please include as a Table the shows the multicollinearity assumption with its mean of VIF test of the variables in supplementary materials?

6. Please approximate the results of tables of the analysis in to 3 digits of the result section? like 0.002, 1.12 etc.…

**********

what does this mean?). If published, this will include your full peer review and any attached files.

**Do you want your identity to be public for this peer review?** If you choose “no”, your identity will remain anonymous but your review may still be made public.

For information about this choice, including consent withdrawal, please see our Privacy Policy

Reviewer #2: No

**********

[NOTE: If reviewer comments were submitted as an attachment file, they will be attached to this email and accessible via the submission site. Please log into your account, locate the manuscript record, and check for the action link "View Attachments". If this link does not appear, there are no attachment files.]

Attachment Submitted filename: comment.pdf

# Author comment: Author response to Decision Letter 2

**DOI:** 10.1371/journal.pgph.0007387.r004

16 Dec 2025

Attachment Submitted filename: Rebuttal_Letter_2.docx

# Peer review: Decision Letter 2

**DOI:** 10.1371/journal.pgph.0007387.r005

**Authors:** Collins Otieno Asweto

21 Jan 2026

PGPH-D-25-01028R2

Co-occurrence of Anaemia and Malaria Among Children Under Five in Uganda: A Joint Modeling Analysis

PLOS Global Public Health

Dear Grace,

Thank you for submitting your manuscript to PLOS Global Public Health. After careful consideration, we feel that it has merit but does not fully meet PLOS Global Public Health’s publication criteria as it currently stands. Therefore, we invite you to submit a revised version of the manuscript that addresses the points raised during the review process.

Please submit your revised manuscript by 19th February, 2026. If you will need more time than this to complete your revisions, please reply to this message or contact the journal office at globalpubhealth@plos.org. When you're ready to submit your revision, log on to https://www.editorialmanager.com/pgph/ and select the 'Submissions Needing Revision' folder to locate your manuscript file.

Please include the following items when submitting your revised manuscript:

- A letter that responds to each point raised by the editor and reviewer(s). You should upload this letter as a separate file labeled 'Response to Reviewers'.
- A marked-up copy of your manuscript that highlights changes made to the original version. You should upload this as a separate file labeled 'Revised Manuscript with Track Changes'.
- An unmarked version of your revised paper without tracked changes. You should upload this as a separate file labeled 'Manuscript'.

Guidelines for resubmitting your figure files are available below the reviewer comments at the end of this letter.

We look forward to receiving your revised manuscript.

Kind regards,

Collins Otieno Asweto, PhD

Academic Editor

PLOS Global Public Health

Journal Requirements:

1. If the reviewer comments include a recommendation to cite specific previously published works, please review and evaluate these publications to determine whether they are relevant and should be cited. There is no requirement to cite these works unless the editor has indicated otherwise.

2. Please review your reference list to ensure that it is complete and correct. If you have cited papers that have been retracted, please include the rationale for doing so in the manuscript text, or remove these references and replace them with relevant current references. Any changes to the reference list should be mentioned in the rebuttal letter that accompanies your revised manuscript. If you need to cite a retracted article, indicate the article’s retracted status in the References list and also include a citation and full reference for the retraction notice.

Additional Editor Comments (if provided):

[Note: HTML markup is below. Please do not edit.]

Reviewers' comments:

Reviewer’s Responses to Questions

**Comments to the Author**

Reviewer #3: All comments have been addressed

Reviewer #4: (No Response)

Reviewer #5: All comments have been addressed

Reviewer #6: All comments have been addressed

Reviewer #7: All comments have been addressed

**********

publication criteria?

Reviewer #3: Partly

Reviewer #4: Yes

Reviewer #5: Yes

Reviewer #6: Yes

Reviewer #7: Yes

**********

3. Has the statistical analysis been performed appropriately and rigorously?

Reviewer #3: Yes

Reviewer #4: Yes

Reviewer #5: Yes

Reviewer #6: Yes

Reviewer #7: Yes

**********

4. Have the authors made all data underlying the findings in their manuscript fully available (please refer to the Data Availability Statement at the start of the manuscript PDF file)?

The PLOS Data policy

Reviewer #3: Yes

Reviewer #4: Yes

Reviewer #5: Yes

Reviewer #6: Yes

Reviewer #7: Yes

**********

5. Is the manuscript presented in an intelligible fashion and written in standard English?

Reviewer #3: Yes

Reviewer #4: Yes

Reviewer #5: Yes

Reviewer #6: Yes

Reviewer #7: Yes

**********

Reviewer #3: The manuscript addresses a highly important topic, but its expression is severely lacking. It suffers from excessive length, causing boredom and distraction for the reader, in addition to repetition. Furthermore, much of the information is unnecessary and contains tedious details. Therefore, in scientific research, repetition and anything that might lead to boredom should be avoided. Please rewrite the manuscript to align with the comments included in the attached file.

Reviewer #4: The manuscript is methodologically sound, with its innovative approach. This is a steppingstone in the region for similar research and analysis of periodically available data particularly targeting children and mothers. However, in this manuscript apart from those mentioned in the attached, it would be great for authors to consider and check the smooth transition between background, methodology, results and discussions.

Reviewer #5: (No Response)

Reviewer #6: (No Response)

Reviewer #7: This manuscript applies a joint Gaussian copula–based bivariate probit model to anemia and malaria outcomes in Ugandan children and addresses an important methodological and public health question. However, several issues need to be addressed to improve epidemiologic validity, interpretability, and consistency with established malaria–anemia biology.

Major comments

1) Internal consistency and interpretation of prevalence/comorbidity (Table 2):

Table 2 reports “anemia only” 43.0%, “malaria only” 29.6%, and “both” 27.4%, summing to ~100%. This implies essentially 0% of children had neither anemia nor malaria, which is highly implausible and requires clarification of the denominator and definitions. Please provide the full 2×2 table including the “neither” category and clearly state the denominator used. As written, these figures also imply P(anemia | malaria) ≈ 48%, which appears unexpectedly low for malaria-endemic settings.

2) External validity: co-occurrence appears lower than expected given endemic biology and benchmarks:

While overall anemia prevalence around ~70% can be plausible in high-burden settings, the implied overlap (e.g., anemia among malaria-positive children) appears low relative to published data from high-burden African settings and Uganda. The Discussion should explicitly compare the study’s conditional prevalences (e.g., P(anemia|malaria), and P(malaria|anemia)) to external benchmarks and explain potential reasons for divergence (case definitions, diagnostic modality, temporal mismatch between infection and anemia, geography, improvements in malaria control, or competing causes of anemia such as nutrition/helminths).

3) MIS measurement constraints should be stated clearly as major interpretive limitations:

Because this is a secondary analysis of MIS data, key measurement constraints should be explicit in Methods and Limitations, including:

hemoglobin measured only in children 6–59 months (excluding 0–5 months),

malaria defined using microscopy vs. RDT (with potential sensitivity differences and temporal issues),

cross-sectional timing (anemia can persist after malaria clearance), and

potential selection bias if severely ill children were less likely to be captured in household surveys.

4) Complex survey design handling in the joint model:

The manuscript notes that descriptive estimates account for MIS survey design, but the joint copula model appears unweighted / not fully design-based. Please clearly state how weights, clustering, and stratification were handled in the joint model, and provide sensitivity analyses (or robust SEs / alternative approaches) to demonstrate that inferences are not materially biased by ignoring complex survey design. If the modeling framework cannot accommodate survey weights, this should be highlighted as a limitation and key results should be shown alongside survey-weighted benchmarks.

5) Interpretation of the dependence parameter (ρ) and unmeasured determinants:

The estimated copula correlation (ρ ≈ 0.19) reflects latent residual dependence after covariate adjustment and should be interpreted accordingly. The Discussion should address why ρ is modest despite strong malaria→anemia pathophysiology (possible dilution by non-malarial anemia, age-pattern differences, measurement limitations). Additionally, common hemoglobinopathies in Uganda (e.g., sickle cell disease/trait, α-thalassemia) can influence hemoglobin and malaria outcomes and are not captured in MIS; this should be acknowledged as an important unmeasured factor potentially contributing to the latent dependence.

6) Causal language / directionality:

Please soften statements implying bidirectional causality (e.g., “illnesses don’t arise independently”). Malaria is a direct cause of anemia, while anemia/nutritional status may also modify susceptibility and care-seeking; however, cross-sectional data cannot establish directionality. The Discussion should reflect association/dependence rather than causation.

Minor / specific comments

Please confirm the hemoglobin cutoff used to define anemia (e.g., Hb <11 g/dL for children 6–59 months) and whether hemoglobin was altitude-adjusted.

Consider reporting anemia severity categories (mild/moderate/severe) if available, since malaria-associated anemia is often more severe and clinically meaningful.

The Methods note species determination from thin smears, but results treat malaria as binary. If available, report species distribution (especially P. falciparum) and comment on biological plausibility.

The reversal/attenuation of the maternal anemia association under joint modeling is interesting; consider adding a short mechanistic interpretation (residual confounding, care-seeking, nutrition, etc.).

Comment briefly on generalizability across regions and seasons; Uganda has strong geographic heterogeneity in malaria and anemia burdens.

Editorial: ensure consistent spelling (anemia/anaemia), fix typos (Microscopy), and define acronyms at first use.

**********

what does this mean?). If published, this will include your full peer review and any attached files.

**Do you want your identity to be public for this peer review?** If you choose “no”, your identity will remain anonymous but your review may still be made public.

For information about this choice, including consent withdrawal, please see our Privacy Policy

Reviewer #3: **Yes:**  Dr. Zuhair Dardona

Reviewer #4: **Yes:**  Dr Adamu Zerihun Gelaw

Reviewer #5: No

Reviewer #6: **Yes:**  Stanley Chinedu Eneh

Reviewer #7: **Yes:**  Baran Erdik, MD, MHPA

**********

[NOTE: If reviewer comments were submitted as an attachment file, they will be attached to this email and accessible via the submission site. Please log into your account, locate the manuscript record, and check for the action link "View Attachments". If this link does not appear, there are no attachment files.]

Attachment Submitted filename: Note on the manuscript entitled.pdf

Attachment Submitted filename: Reviewer Comments from Dr Adamu Gelaw.docx

Attachment Submitted filename: PGPH-D-25-01028 review.docx

# Author comment: Author response to Decision Letter 3

**DOI:** 10.1371/journal.pgph.0007387.r006

9 Feb 2026

Attachment Submitted filename: Rebuttal_Letter_5.docx

# Peer review: Decision Letter 3

**DOI:** 10.1371/journal.pgph.0007387.r007

**Authors:** Collins Otieno Asweto

27 Mar 2026

PGPH-D-25-01028R3

Co-occurrence of Anaemia and Malaria Among Children Under Five in Uganda: A Joint Modeling Analysis

PLOS Global Public Health

Dear Grace,

Thank you for submitting your manuscript to PLOS Global Public Health. After careful consideration, we feel that it has merit but does not fully meet PLOS Global Public Health’s publication criteria as it currently stands. Therefore, we invite you to submit a revised version of the manuscript that addresses the points raised during the review process.

Please submit your revised manuscript by 24/04/2026. If you will need more time than this to complete your revisions, please reply to this message or contact the journal office at globalpubhealth@plos.org. When you're ready to submit your revision, log on to https://www.editorialmanager.com/pgph/ and select the 'Submissions Needing Revision' folder to locate your manuscript file.

Please include the following items when submitting your revised manuscript:

- A letter that responds to each point raised by the editor and reviewer(s). You should upload this letter as a separate file labeled 'Response to Reviewers'.
- A marked-up copy of your manuscript that highlights changes made to the original version. You should upload this as a separate file labeled 'Revised Manuscript with Track Changes'.
- An unmarked version of your revised paper without tracked changes. You should upload this as a separate file labeled 'Manuscript'.

Guidelines for resubmitting your figure files are available below the reviewer comments at the end of this letter.

As the corresponding author, your ORCID iD is verified in the submission system and will appear in the published article. PLOS supports the use of ORCID, and we encourage all coauthors to register for an ORCID iD and use it as well. Please encourage your coauthors to verify their ORCID iD within the submission system before final acceptance, as unverified ORCID iDs will not appear in the published article. *Only* the individual author can complete the verification step; PLOS staff *cannot*  verify ORCID iDs on behalf of authors.

We look forward to receiving your revised manuscript.

Kind regards,

Collins Otieno Asweto, PhD

Academic Editor

PLOS Global Public Health

Journal Requirements:

If the reviewer comments include a recommendation to cite specific previously published works, please review and evaluate these publications to determine whether they are relevant and should be cited. There is no requirement to cite these works unless the editor has indicated otherwise.

Please review your reference list to ensure that it is complete and correct. If you have cited papers that have been retracted, please include the rationale for doing so in the manuscript text, or remove these references and replace them with relevant current references. Any changes to the reference list should be mentioned in the rebuttal letter that accompanies your revised manuscript. If you need to cite a retracted article, indicate the article’s retracted status in the References list and also include a citation and full reference for the retraction notice.

Reviewers' comments:

Reviewer’s Responses to Questions

**Comments to the Author**

Reviewer #3: (No Response)

Reviewer #4: All comments have been addressed

**********

publication criteria?

Reviewer #3: Partly

Reviewer #4: Yes

**********

3. Has the statistical analysis been performed appropriately and rigorously?

Reviewer #3: Yes

Reviewer #4: Yes

**********

4. Have the authors made all data underlying the findings in their manuscript fully available (please refer to the Data Availability Statement at the start of the manuscript PDF file)?

The PLOS Data policy

Reviewer #3: Yes

Reviewer #4: Yes

**********

5. Is the manuscript presented in an intelligible fashion and written in standard English?

Reviewer #3: Yes

Reviewer #4: Yes

**********

Reviewer #3: Dear Authors,

In the revised manuscript, however, most of the changes suggested in the previous review were not made and several important issues are still not sufficiently revised. I therefore have attached another file with my additional comments and proposed revisions. I urge you to revise your manuscript taking into account my comments and suggested changes and especially greatly improve the Results and Discussion sections of your manuscript as these sections still need the most improvements with respect to how the data are presented and interpreted. Some parts of the methodology also need some changes and additional justification to avoid some of the issues mentioned in the previous review. I hope you will perform a major revision of your manuscript as suggested and take into account the issues with the methodology with the aim to improve the scientific quality of your manuscript with respect to the presentation and interpretation of your results.

I wish you all the best with revising your manuscript.

Best regards.

Reviewer #4: This has been an incredible work, the authors have exhibited a high degree of commitment, and effort in revising this manuscript. Looking into each and every details, the comments have been carefully addressed with an open and meticulous attention from the beginning to the end. The author genuine commitment in improving the work as per the comments provided with details of review for ensuring and addressing clarity, rigor, and in accordance to the journal standard is clear and commendable.

From my first review perspective, the resubmitted manuscript appears well scrutinized, methodologically sound, and comprehensively articulated. The initial gaps seen precisely addressed, and the innovative joint modeling application (use of a Gaussian copula approach) has been touch based to be suitable for the research paper and problem under investigation.

Furthermore, the authors have successfully presented sophisticated statistical concepts in a manner that is suitable and relevant to public health practice. Most importantly, the research paper will be able to provide critical insights on the interdependence of the two diseases (Anemia & Malaria) among children aged under five Years.

In general, I definitely commend the work done, and the authors for their professionalism, their time taken, their detailed review, and high degree of commitment in revising every aspect of the work. In due course of time, it is my strong view that the work meets the PLOS Global Public Health’s publication criteria and will continue to make a valuable contribution to the literature on public health in the context of children comorbidities. I am very satisfied with the quality of the work and would be glad to see it published in its entirety without any conditions. And, it is a pleasure to have an opportunity to review the paper.

**********

what does this mean?). If published, this will include your full peer review and any attached files.

**Do you want your identity to be public for this peer review?** If you choose “no”, your identity will remain anonymous but your review may still be made public.

For information about this choice, including consent withdrawal, please see our Privacy Policy

Reviewer #3: **Yes:**  Dr. Zuhair Dardona

Reviewer #4: **Yes:**  Dr Adamu Zerihun Gelaw

**********

[NOTE: If reviewer comments were submitted as an attachment file, they will be attached to this email and accessible via the submission site. Please log into your account, locate the manuscript record, and check for the action link "View Attachments". If this link does not appear, there are no attachment files.]

Attachment Submitted filename: Note on the manuscript entitled New.pdf

# Author comment: Author response to Decision Letter 4

**DOI:** 10.1371/journal.pgph.0007387.r008

26 Apr 2026

Attachment Submitted filename: Rebuttal_Letter_7.docx

# Peer review: Decision Letter 4

**DOI:** 10.1371/journal.pgph.0007387.r009

**Authors:** Jen Edwards

10 Aug 2026

PGPH-D-25-01028R4

Co-occurrence of Anaemia and Malaria Among Children Under Five in Uganda: A Joint Modeling Analysis

PLOS Global Public Health

Dear Dr. Kakaire,

Thank you for submitting your manuscript to PLOS Global Public Health. After careful consideration, we feel that it has merit but does not fully meet PLOS Global Public Health’s publication criteria as it currently stands. Therefore, we invite you to submit a revised version of the manuscript that addresses the points raised during the review process.

The manuscript has been evaluated by one reviewer and their comments are available below. They request some minor editorial changes to the manuscript at this stage. Could you please revise the manuscript to carefully address the concerns raised?

Please submit your revised manuscript by Sep 06 2026 11:59PM. If you will need more time than this to complete your revisions, please reply to this message or contact the journal office at globalpubhealth@plos.org. When you're ready to submit your revision, log on to https://www.editorialmanager.com/pgph/ and select the 'Submissions Needing Revision' folder to locate your manuscript file.

Please include the following items when submitting your revised manuscript:

- A letter that responds to each point raised by the editor and reviewer(s). You should upload this letter as a separate file labeled 'Response to Reviewers'.
- A marked-up copy of your manuscript that highlights changes made to the original version. You should upload this as a separate file labeled 'Revised Manuscript with Track Changes'.
- An unmarked version of your revised paper without tracked changes. You should upload this as a separate file labeled 'Manuscript'.

Guidelines for resubmitting your figure files are available below the reviewer comments at the end of this letter.

As the corresponding author, your ORCID iD is verified in the submission system and will appear in the published article. PLOS supports the use of ORCID, and we encourage all coauthors to register for an ORCID iD and use it as well. Please encourage your coauthors to verify their ORCID iD within the submission system before final acceptance, as unverified ORCID iDs will not appear in the published article. *Only* the individual author can complete the verification step; PLOS staff *cannot*  verify ORCID iDs on behalf of authors.

We look forward to receiving your revised manuscript.

Kind regards,

Jen Edwards

Staff Editor

PLOS Global Public Health

Journal Requirements:

1. If the reviewer comments include a recommendation to cite specific previously published works, please review and evaluate these publications to determine whether they are relevant and should be cited. There is no requirement to cite these works unless the editor has indicated otherwise.

2. Please review your reference list to ensure that it is complete and correct. If you have cited papers that have been retracted, please include the rationale for doing so in the manuscript text, or remove these references and replace them with relevant current references. Any changes to the reference list should be mentioned in the rebuttal letter that accompanies your revised manuscript. If you need to cite a retracted article, indicate the article’s retracted status in the References list and also include a citation and full reference for the retraction notice.

Additional Editor Comments (if provided):

[Note: HTML markup is below. Please do not edit.]

Reviewers' comments:

Reviewer’s Responses to Questions

**Comments to the Author**

Reviewer #8: All comments have been addressed

**********

publication criteria?

Reviewer #8: Yes

**********

3. Has the statistical analysis been performed appropriately and rigorously?

Reviewer #8: Yes

**********

4. Have the authors made all data underlying the findings in their manuscript fully available (please refer to the Data Availability Statement at the start of the manuscript PDF file)?

The PLOS Data policy

Reviewer #8: Yes

**********

5. Is the manuscript presented in an intelligible fashion and written in standard English?

Reviewer #8: Yes

**********

Reviewer #8: This is a "must-read" for researchers looking to bridge the gap between dynamical systems theory and empirical surveillance data.

**********

what does this mean?). If published, this will include your full peer review and any attached files.

**Do you want your identity to be public for this peer review?** If you choose “no”, your identity will remain anonymous but your review may still be made public.

For information about this choice, including consent withdrawal, please see our Privacy Policy

Reviewer #8: **Yes:**  Nicholas Siame Adam

**********

[NOTE: If reviewer comments were submitted as an attachment file, they will be attached to this email and accessible via the submission site. Please log into your account, locate the manuscript record, and check for the action link "View Attachments". If this link does not appear, there are no attachment files.]

Attachment Submitted filename: Reviewer Response to PGPH-D-25-01028_R4.docx

# Author comment: Author response to Decision Letter 5

**DOI:** 10.1371/journal.pgph.0007387.r010

4 Sep 2026

Attachment Submitted filename: Rebuttal_Letter_8.docx

# Editor report: Decision Letter 5

**DOI:** 10.1371/journal.pgph.0007387.r011

**Authors:** Julia Robinson

13 Sep 2026

Co-occurrence of Anaemia and Malaria Among Children Under Five in Uganda: A Joint Modeling Analysis

PGPH-D-25-01028R5

Dear Mr Kakaire,

We are pleased to inform you that your manuscript 'Co-occurrence of Anaemia and Malaria Among Children Under Five in Uganda: A Joint Modeling Analysis' has been provisionally accepted for publication in PLOS Global Public Health.

Before your manuscript can be formally accepted you will need to complete some formatting changes, which you will receive in a follow up email. A member of our team will be in touch with a set of requests.

Please note that your manuscript will not be scheduled for publication until you have made the required changes, so a swift response is appreciated.

IMPORTANT: The editorial review process is now complete. PLOS will only permit corrections to spelling, formatting or significant scientific errors from this point onwards. Requests for major changes, or any which affect the scientific understanding of your work, will cause delays to the publication date of your manuscript.

If your institution or institutions have a press office, please notify them about your upcoming paper to help maximize its impact. If they'll be preparing press materials, please inform our press team as soon as possible -- no later than 48 hours after receiving the formal acceptance. Your manuscript will remain under strict press embargo until 2 pm Eastern Time on the date of publication. For more information, please contact globalpubhealth@plos.org.

Thank you again for supporting Open Access publishing; we are looking forward to publishing your work in PLOS Global Public Health.

Best regards,

Julia Robinson

Executive Editor

PLOS Global Public Health

***********************************************************

Reviewer Comments (if any, and for reference):
